# St. Thomas's Hospital

**Published:** 1831-04-01

**Authors:** 


### ST. THOMAS'S HOSPITAL.

### I. RHEUMATIC AFFECTION OF THE CHEST —DIAGNOSIS BETWEEN IT AND PLEURITIS. By Dr. ELLIOTSON.

IN a late clinical lecture on pleuritis, Dr. Elliotson introduced a case of rheumatic inflammation of the parietes of the chest, by way of comparison or contrast with phlogosis of the pleura—a comparison which is not useless, since the one disease is very often confounded with the other, to the detriment of the patient, by the unnecessary effusion of blood thereby occasioned.

CASE. William Key, æt. 19, in William's ward, had been ill a fortnight. At first he had rheumatism of the left knee, and he now and then has it there still; but he has it particularly about the left acromion, all over the same shoulder, clavicle, and front of the chest. There is great pain of the chest, and considerable tenderness on pressure; and great pain on inspiration, but no cough and no expectoration. Now the existence of pain in the chest, rendered severely cutting on inspiration, might give the idea that the person was labouring under pleuritis. The two diseases, however, are very easily distinguished by any one who has seen rheumatism of the chest before. The diagnosis was this: the pain was not confined to one spot, but more or less diffused over the front of the chest generally; it is rare for the pain of pleuritis to be so diffused. In the next place, there was great heat of the part: in active rheumatism there is generally heat of the part, whether it be situated in a joint or in any other portion of the body. Farther, the pain was increased by the very slightest pressure, such as could not influence the pleura; and not merely between the ribs, but on them—even on the sternum, where the pleura, again, could not be influenced. In pleuritis the pressure must be between the ribs, or if on the ribs must be very strong to give pain, unless in the most violent cases, where the whole parietes suffer as well as the pleura.

The pressure which produced pain in the woman was between the ribs, but in this man the least pressure on the ribs themselves, or even upon the sternum, produced great distress. Fourthly, rheumatism existed also in other parts. From the combination of all these symptoms, particularly from the third, I inferred the nature of the disease. The heat and the diffused pain alone would not have shewn the nature of the disease, because sometimes in pleuritis both those symptoms are present. The fact of rheumatism being in the shoulder would not alone have been decisive, because in some cases of pleuritis we have pain also about the axilla. Nay, the rheumatism in the knees, or other parts, would not have been decisive, because a person may have both pleuritis and rheumatism. The same cause that produces the one disease may produce the other, and is commonly in both—the application of cold, especially with moisture, and when the body is overheated. Taking, however, all these symptoms conjointly, there could be no doubt about the disease. He had no cough, no expectoration—and this still farther illustrated the true nature of the disease; though in some instances of severe rheumatism of the chest there is accidentally a very slight catarrhal affection, which produces a little expectoration—just as much as is often seen in pleuritis. The man was not ordered to bed, and he shewed no distress of countenance; yet in some cases of rheumatism of the chest I have witnessed such indisposition as to confine the patient to bed: for acute rheumatism any where, and therefore in the chest, may occasion this; and I have seen the pain on breathing so great as to occasion much anxiety of countenance.

The *treatment* of the two diseases would be exactly the same in principle; but in the case of rheumatism there would be no occasion for the adoption of such active measures as in the case of pleurisy. Whether a nice diagnosis is necessary or not in any disease, it should invariably be made, because we should always practise our art in the best manner; but there are two reasons why it is important to make a careful diagnosis here: in the first place, in order that you may be enabled to inform the patient and his friends whether there is danger or not—pleurisy being dangerous, while rheumatism is not, except it attack some vital part; and secondly, because, although you employ the same measures in the two diseases, it is not requisite in rheumatism to employ them to the same extent. I ordered no general bleeding for this man, but thirty leeches to be applied on the front of his chest; and as he had rheumatic pains about the shoulder and other parts of the body, I judged constitutional measures requisite—gave him five grains of calomel night and morning. The leeches completely relieved the front of the chest, but the next day I found the same pain in the opposite shoulder (this is the character of acute rheumatism, to leave one part and fly to another), and I ordered the leeches to be applied there. On the following day I found the rheumatism in the neck, and I had leeches applied to that part, and to-day found him infinitely better. He had gone out of doors contrary to my wishes, and has a little sore throat, and still pain in the right shoulder; but the chest is liberated from the disease, and there is little now the matter with him.*

In a preceding part of the same clinical lecture Dr. Elliotson details minutely a case of pleuritis, and, in fact, gives a dissertation on the complaint, delineating its symptoms as revealed by the common phenomena presented to the eye, and the auscultic indications drawn from the stethoscope. We are glad to see auscultation thus publicly taught in one of our greatest hospitals. Ten years ago we would have been laughed at, if we had predicted such an event. What! That wooden bauble! That tom-foolery! That piece of QUACKERY, introduced into a lecture by any respectable physician! Oh no! the thing is impossible.

* Med. Gazette.

II. TWO CASES WHERE DEATH WAS OCCASIONED BY EXCESSIVE LOSS OF BLOOD. DR. ELLIOTSON.

In a clinical lecture delivered by the above talented physician, as reported in the MEDICAL GAZETTE, we find the following cases, which we shall separate from the commentaries made on them.

CASE 1. "Abraham Dick, æt. 39, a bargeman, in consequence of violent pains of the head and epistaxis for the preceding fortnight, was cupped at the back of the neck, and between the shoulders, on the 21st ult. with great relief. The day after the cupping, during a fit of vomiting, the scarifications began to bleed afresh, and hæmorrhage ensued to such an amount, in spite of all efforts to stop it, that a very large quantity of blood was lost, and he was therefore brought to the hospital on the 26th, labouring under its effects. There can be no question about the propriety of the cupping, for he had vertigo, drowsiness, violent pain of the head, and the cupping relieved all the symptoms; so that it was the accidental circumstance of hæmorrhage after the bleeding that did the mischief. It appeared that besides this affection he had been subject to vomiting, and likewise to a little cough, but particularly to fits of vomiting, which most likely depended upon the state of the head. The vomiting, however, by the bleeding grew worse, and every thing he took was rejected from the stomach, the act of vomiting being attended with considerable pain. The countenance of the patient corresponded with the effect produced by loss of blood; he was pale, straw-coloured, and complained of great debility, unable to stand or walk; his pulse was low and feeble; he likewise complained of great thirst, which is a common occurrence when there has been any great loss of the fluids either by sweating, purging, great flow of urine, or blood-letting. He had now no pain of the head, but on sitting up or moving about there was giddiness; this, however, passed off the moment he was laid down. He felt chilly, and sometimes almost fainted; he was also restless and anxious, and his hands were tremulous when I first saw him. On feeling his pulse while he reclined, and also after making him sit up, I found there was a considerable difference, for the moment he sat up the pulse became weak and irregular, but as soon as he again was laid down, it became fuller and regular. I repeated this experiment, and found the same effect; but on a third trial the same circumstance was not observed.

He came in after I had made my visit on the evening of the 26th of October, and he was very properly ordered laudanum and good nourishment. He took thirty minims of tincture of opium, and he was ordered two pints of beef-tea, and two of milk. The case was not one of great intensity, but was decidedly a case of the ill-effects of the loss of blood. He was likewise ordered a tonic, certainly one of the best in restoring the system when it has lost an abundance of blood—iron, and in the form of the subcarbonate. This will not act quickly; so that if you want to produce an immediate effect this would not be a proper remedy. As in the present case, however, there was no immediate urgency, it was very proper to administer the iron, any immediate benefit being intended to be derived from the opium. At his admission the scarifications were bleeding, but the flow was arrested by pressure. On the 27th it was found that he still vomited; that all his food was rejected; and that he had great pain in the scrobiculus cordis at the moment of vomiting, but at no other time. The pulse was said to be 88 and full, and there was thirst. He appeared to revive at night, but at four o'clock in the morning he coughed, and the hæmorrhage was renewed to such a degree that it was necessary to call the dresser, who again stopped it by pressure. At noon, the vomiting being no better, half a grain of opium was prescribed in substance in the room of the tincture, which had been rejected from his stomach. This quantity of solid opium was ordered to be given every four hours, and from the first dose the vomiting was in some measure checked, and the opium was no longer rejected. He slept that night, and on the following day it appeared that he had vomited only four times during the twenty-four hours, and that was on coughing or taking food, which is a common circumstance when persons have been subject to severe vomiting. There was less pain when he vomited, and less tenderness also in the epigastric region; it appeared, therefore, that there was rather morbid irritability than an inflammatory condition of the stomach. He still complained of giddiness, but his headache was now but slight; his pulse was 80, full, and rather sharp; hands still tremulous. On the 29th he had slept better, and had only vomited once, shewing the propriety of the treatment. If the vomiting had been supposed to be inflammatory, and there had been considerable and constant tenderness on pressure, leeches would have been applied, but that would have made him worse; whereas, by giving him opium, he was, so far as the vomiting was concerned, better. On the 28th, as he complained of want of sleep, I preferred a full dose to the smaller ones, and substituted for them three grains at once at bed-time, and I allowed him ℥iv. of wine in the 24 hours. A clyster was required on the 29th: I found that he had vomited but once, had slept better, felt stronger. The three grains of opium, the wine, strong beef-tea, milk, and iron, were ordered to be continued daily. On the 30th the report is that he had been rather restless, and complained of a good deal of giddiness; his bowels had been opened by a clyster, which has made him feel better; he had vomited only four times the last 24 hours, and that was when he coughed; the pulse was softer. On the 31st he had passed a very bad night through great restlessness and anxiety. He had also wandered in his conversation, and attempted to leave his bed. At two o'clock, A. M. his nose began to bleed, and continued to do so at intervals till six in the morning, though measures were used to stop it; he lost, however, not more than two or three ounces of blood. At midnight his pulse was very variaable at one time, and seemed to be rather full and compressible, and at another time it was almost indistinct. The sister of the ward said that fits of palpitation of the heart came on so violently, that they caused the bed to shake, and that his breathing was performed with a great noise, like that of croop, during his sleep. I presume it was stertorous. He was given half a drachm of liquor ammoniæ subcarbonatis every three hours, and three ounces of brandy at intervals, by which he was much relieved, and towards morning he was considerably better. But on visiting him at four o'clock on Sunday afternoon his countenance was still very anxious, and he wandered in his conversation; there was rather more tremor in his hands, and the pulse was sharp and variable, sometimes being a mere thread. At nine o'clock in the evening he was lying perfectly insensible, his eyes fixed, his pupils contracted, his pulse slow and feeble, and respiration taking place at long intervals. He died at half-past ten o'clock."

There was no dissection.

*Case* 2. "The other case which I shall mention occurred among the women, and the affection of the head from the loss of blood proceeded to actual convulsions, and considerable difficulty must have been experienced in ascertaining the nature of the case. This woman was 19 years of age, and had had two children. She was brought to the hospital on Thursday in a state of insensibility and convulsions, and it appeared that she had been delivered of a child seven weeks before. She was in convulsions of an irregular character, with insensibility and stertorous breathing. She was seen and prescribed for after my visit: 16 ounces of blood were taken from the back of her neck, and 15 grains of extract. coloc. c. was administered, together with an injection, and a cold lotion was applied to her head. No information was given respecting the history of the case: all that was said by the friends was, that she had been seized with convulsions that morning, and became insensible. The natural conclusion certainly was, that this was a case of determination of blood to the head, and the proper indication of cure was to take away blood from the head. The head, too, was hot; and had she been prescribed for by myself, I have no hesitation in saying that I think it more than likely that I should have treated her in the same way. Her pulse was full; she had lain-in seven weeks; the head was hot; she was in a state of insensibility and convulsions, and the breathing was stertorous, and had she died that day without being cupped — without having blood taken from the head—I think had I been the person I should have blamed myself. But it turned out, when more of her history was known, that the propriety of the treatment was doubtful. When I first saw her the following morning she was in a state of convulsion, with moaning, stupor, and stertorous breathing. Luckily for me, the gentleman who had attended her in her confinement had come to the hospital to see her, and he informed me that she had had excessive flooding in her labour, and that it had been necessary to turn the child, and that at the time of this hæmorrhage she had had a degree of convulsions similar to those now present. He also told me that, although seven weeks had elapsed, she had never recovered her colour, and I certainly found her of a deadly white; but notwithstanding that appearance, had I seen her the day before, I should have considered her as a person in an epileptic state, with great stupor, and a tendency to apoplexy, and ascribed the paleness to the epilepsy, in which I have often seen persons ghastly and pale. I found her pulse full, and I should have thought from first feeling it that it justified me in taking blood, at least from her head, had I not been informed by this gentleman that she had had previous flooding, and had I not noticed that besides the fulness the pulse had a peculiar sharpness; certainly it was a hæmorrhagic sharpness, but it was likewise so full as to make me hesitate for a moment, and consider whether I ought not to apply leeches to the head. However, while I was standing at the bed-side considering the history of her case, and taking all the circumstances connected with it into consideration, the character of the pulse altered, it actually became a little irregular and decidedly weaker, and I very soon saw clearly that it was a case of exhaustion — convulsions from loss of blood.

The treatment to be adopted was at once indicated, namely, to give stimuli and nourishment. I administered, at first, twenty minims of liquor ammoniæ in camphor mixture, and on watching its effects I perceived that it scarcely stimulated her. I repeated it in about 20 minutes, with half a drachm of tincture of opium. I waited perhaps 20 minutes more, and during the whole of this time the pulse was regularly sinking, becoming weaker and weaker, and losing its sharpness: she became colder, and a difficulty of swallowing supervened, so that nothing could be taken into her mouth. I sent for the stomach-pump; but as the stomach, being very weak in these cases, frequently becomes so irritable as to reject nourishment, and render the subsequent administration of medicine and other stimulants fruitless, I attempted to nourish her per rectum, and a quantity of strong beef tea, with four eggs beat up in it, was thrown up the intestine; but the whole was immediately rejected; it was scarcely thrown in before it was discharged. Under these circumstances I at once employed the stomach-pump, and by its means some brandy, wine, and more laudanum, were got into the stomach. I staid in the hospital some few hours, and in the course of that time a considerable quantity of brandy was got down; but she regularly sunk, none of the stimulants making any impression upon her, except in one instance, when the pulse rallied, though in a very slight degree, for a few minutes. With that exception the decline of life was steady and progressive, the breathing became slower, and in the afternoon, about four o'clock, she expired."

No dissection of this case could be obtained, which is the more to be regretted, as we apprehend it would have shewn some curious appearances. But we do not consider ourselves justifiable in stating more than the bare facts, leaving our readers to judge for themselves.

### ST. THOMAS'S HOSPITAL.

### DR. ELLIOTSON ON DROPSY.

IN a clinical lecture delivered by Dr. E. on the 22d of November last, we find the following observations, as published in our cotemporary, the Medical Gazette.

"The only case of interest was one of severe general dropsy, which was cured; and certainly it was a case of very considerable interest. It occurred in a girl named Maria Sudgwick, æt. 14, and admitted on the 14th October. She was delicate, had light hair, a fair and very fine skin, and ruddy complexion. After the disease was removed, the redness in the cheeks remained, so that it was her natural colour. It appeared that she had been the subject of ague at different times for the last three years; that about three months before her admission her belly had become swelled, and not long afterwards her legs; there had been also cough for about a month, but she had no pain in any part of the chest. She was swollen when I saw her from head to foot, but the abdomen was particularly swelled. The right eye was quite closed on account of the swelling of the face, and the left nearly so. The whole of the abdomen was tender on pressure, and the respiration was quick when she lay on her back; but that in all probability arose from the tenderness of the abdomen. She had, besides this, diarrhœa—diarrhœa attended by griping. Between the great distention of the abdomen by fluid, and its tenderness, it was impossible for me to ascertain whether there was any enlargement or induration of the liver or of the spleen; nor could I ascertain whether indeed any particular organs were inflamed.

The case appeared a very bad one, for there was besides intense general dropsy, anasarca, and ascites, and extreme tenderness of the whole abdomen, a great feebleness of the pulse—it was quick, but exceedingly feeble. It was very possible too that she might have great organic disease, as she had suffered from ague.

The indication of treatment, however, was of course to subdue the inflammatory state of the peritoneum in the first instance. I dreaded the application even of leeches, from the great smallness and feebleness of the pulse; I ventured to put on twelve, and after their removal I covered the part with a constant bran poultice. Mere smallness of the pulse would have been no counter-indication to free bleeding, had it been also solid; but it was extremely soft and feeble. The leeches relieved the tenderness of the abdomen considerably; the pulse, however, the next day was so much the weaker, and I therefore could not think of applying leeches a second time; yet she felt better. I ordered her, at the same time with the leeches, three grains of hydrarg. c. creta, and the sixth of a grain of opium every four hours, making 18 grains of hydrarg. c. creta and one grain of opium in the 24 hours. It was necessary to give her this form of mercury on account of the diarrhœa, for any other would only have irritated the intestines, and increased the purging: and it was necessary even to guard it with opium, which, independently too of the mercury, would have been proper, on account of the diarrhœa. I gave her this mercury on account of the inflammatory state of the peritoneum—on account of the dropsy being evidently of an inflammatory nature. I gave her no diuretics. The treatment evidently was to consist in the removal of the inflammatory state of the peritoneum, and checking the diarrhœa; for had the diarrhœa continued, she most probably would have suffered from it considerably, and perhaps have sunk. Yet it would have been wrong to have stopped it suddenly, for the cessation of secretion of the inner membrane of the intestines, might have increased the secretion by the peritoneal coat. I have known ascites produced by a sudden cessation of diarrhœa. The diarrhœa was partially checked, and the tenderness and tension of the abdomen diminished on the very next day. The motions that had occurred were serous, thin, copious, and very offensive. She vomited two or three times the second day after breakfast, and the pulse was scarcely perceptible; yet she did not feel herself weaker. On the 16th (the third day) she felt much better; there was no pain on pressure on any part of the abdomen, still less tension and tumefaction; the vomiting had not returned; the bowels had only been evacuated twice in the last 24 hours; the pulse had become more distinct; the motions, however, which had occurred were copious and watery, but were now yellowish; the quantity of urine was increased, and she slept very well. The treatment was not altered during the whole time that I had her under my care. The leeches of course were not applied again, but the poultice, the hydrarg. c. creta, and likewise the opium, were continued in exactly the same doses for three weeks. Without doing any thing more than this, she gradually became perfectly well; the abdomen subsided to its proper size, the tenderness never returned, the pulse became gradually slower and stronger, the anasarca disappeared from every part of the body, she very soon left her bed, and appeared in perfect health. I was then able to examine the abdomen, and I found no enlargement of any organ—no enlargement nor induration could be discovered in any organ whatever. The case was one very striking, and doubtless many who saw her thought she would die, and I had very great apprehensions myself; but under that simple treatment the result was as I have mentioned. She was presented on the 18th November. Till the 19th of October I allowed here only milk, gruel, arrow-root, and weak broth; but from that time she had half a mutton chop daily, which she continued to take till she went out.

Now, however simple this treatment was, I am convinced that any other would have destroyed. Had I given her common stimulants or tonics, or full diet; or had I given her stimulating diuretics, I should have most probably induced excessive irritation of the mucous membrane of the stomach and intestines—I should have suppressed the urine altogether, have increased the peritonitis, and destroyed her. I have no doubt that had she taken squills and spirit of nitric æther, and been allowed wine, she would have presently sunk. The case was one of inflammatory dropsy, the inflammation being chiefly seated in the peritoneum, and attended with great debility. Had there been no such debility, I should not have been contented with leeches, but have bled her well from the arm; and if I had applied leeches, I should have done so very freely. I should also not have given her even weak broth, but confined her to slops—to barley water and tea.

You are of course well aware that many cases of dropsy are exactly of this description. Dropsies, in my lectures on the practice of medicine, I endeavoured to generalize with many other affections: with fluxes, for example,—discharges from the mucous membrane; with hæmorrhages of all kinds, whether from mucous membranes or not, and with various organic diseases. I stated that these occurrences might be inflammatory, or they might be free from an inflammatory state; that they are all to be diminished very much, and many of them cured simply by the treatment of inflammation, or that mode of treatment may be exceedingly improper. This is most strikingly exemplified in the discharges from the various mucous membranes, very many of which may be cured by simple bleeding, general or local, and starving, while others require stimuli to the part itself, or general stimuli and tonics. Dropsy sometimes arises, therefore, from an inflammatory state, but it is to be remembered that it sometimes arises from an opposite condition of the system. When persons are bled to excess they become dropsical, not in that instance from inflammation, but from debility. When persons are starved, their legs are observed to swell; they have hollowness of the eyes, extreme contraction of all the features, but enlarged ankles. Frequently, too, dropsy arises from an obstruction to the return of the lymph, or if the blood in the veins is impeded; and in the latter case, from the great distention that is produced, the bloodvessels ease themselves by pouring forth fluid into the serous or cellular membranes. Hæmorrhage will sometimes arise from the same cause: it is not uncommon in diseases of the heart, in which the blood is obstructed, for hæmorrhage to occur from the alimentary canal—hæmorrhage which speedily proves fatal. Sometimes dropsy arises without our being well able to explain its origin. When there is disease of the kidney, it is common for dropsy to occur; one can hardly, however, see why it should be so, unless it arise from the quantity of urine being diminished, and the secretions of the cellular and serous membranes making up for the deficiency. Yet frequently there is here no deficiency of urine; we hardly see why, in disease of some other viscera, there should be dropsy unless from the cachectic state induced in the whole system. It is very common in disease of the womb for a female to become anasarcous, even when there has been no great flooding; and it is likewise common in diseases of the spleen and the liver for the same thing to occur. You might suppose, *à priori*, that there is an obstruction to the blood in these instances, but the supposition is often unfounded. Obstruction in the liver and spleen ought rather to induce diarrhœa, or hæmorrhage from the mucous membrane of the alimentary canal. In the case of disease in the kidney, at least, obstruction will not explain it. We cannot explain why the whole body falls into a state of dropsy unless from the whole system becoming cachectic through the renal disease, or the disease of the kidney being a part only of a generally depraved state of the habit.

If the kidney be organically affected, or have great congestion of blood, or an inflammatory state, I believe the urine is generally albuminous; but I do not think that the circumstance of the urine being albuminous is, on the other hand, a proof that the kidney is in this state; at least in a state of organic disease. Because I have seen so many persons cured of dropsy, and restored to perfect health, who had albuminous urine; and if the kidney had been originally diseased, we can hardly suppose that that would have been the case; nor could congestion, and inflammation of the kidney, be supposed, because there were no signs of such affections. I have continually seen albuminous urine in cases of dropsy without any reason, first or last, to suspect disease of the kidney, and I have seen the dropsy completely cured. I think, therefore, that although it is possible that in disease of the kidney, and in congestion of that organ, the urine may generally be albuminous, the converse cannot be said,—that if the urine be albuminous we cannot necessarily conclude that the kidney is in these diseased conditions. When the disease, however, is of the nature that it was in this case—inflammatory, there are sure to be general marks of inflammation, or marks of inflammation of a particular part. I should not have supposed here that there had been a general inflammatory state of the system, for the pulse was exceedingly weak, but there was decidedly inflammation in one part, that is, of the peritoneum.

When dropsy is of an inflammatory nature you will generally see fulness about the head, or an inflammatory state of the chest, or an inflammatory state of the abdomen, and frequently we have all three parts in an inflammatory state. You frequently see the head, from its fulness, become oppressed; the patient complains of drowsiness, or a tightness of the forehead, as though it were bound with hoops, giddiness, or headache; or if you desire him to make a deep inspiration; you find soreness of the chest, and on listening at the parietes you discover a rattle; or if you examine the abdomen you find tenderness some where. These symptoms are sometimes inconsiderable; but you will generally perceive inflammatory affection either of the head, chest, or abdomen: perhaps the patient will not mention them spontaneously, and therefore it is necessary that you should inquire after them."

II. INTERMITTENT HEMIPLEGIA.

Dr. Elliotson has put on record, in one of his clinical lectures, a case of this kind, which is the first he has ever witnessed. Our readers are aware that we have recorded several cases of this description on our pages. We insert the present case for several reasons.

"There was a case of disease of the nervous system presented of a curious character, the first of the kind I ever met with, intermittent palsy. I have read of it in authors, and you will find it mentioned by Cullen as *paralysis intermittens*. Now among all the patients I have ever seen, and these amount to between thirty and forty thousand, including those in various public establishments and private practice, I had never met with an instance of this description. It was a case of intermittent hemiplegia. The man was admitted into Jacob's Ward some time ago, and I mentioned his admission at the time. I gave him no medicine, because I was desirous of seeing whether his account was true or not. I seldom give medicine in aguish or intermittent complaints till some one in the hospital has witnessed the occurrence of the paroxysms. He staid here three weeks without having a paroxysm. He was, however, a very respectable man, and I did not doubt his account. He then went out of the hospital, enjoined by me to return if his disease re-appeared. One day when I came to the hospital, some time afterwards, I found him in the courts, and he said he had been seized with a paroxysm that morning, and he actually was then in a state of hemiplegia of the left side. I saw it myself. I made him walk, and he dragged his leg in a semi-circular way, as patients usually do when they are labouring under hemiplegia, and he could not raise his left arm. It began at ten o'clock, and this was the usual course of the disease. He had told me originally that the paroxysms came on at 10 o'clock in the morning, not every day, but every third or fourth day, and, with a single exception, never after a longer period than that; but on one occasion there was an interval of sixteen days. He was 48 years of age, and had been subject to this affection for two years and a half; and the paroxysm would last from three to four hours. But although it only lasted that time, he was not perfectly clear from it the whole of the day. He never knew the paroxysms begin later than 11 o'clock, or earlier than 10; from 10 to 11 was the regular period, till a week before he had been admitted, when one attack came on at half-past 10 in the evening —the usual hour, but in the evening instead of the morning. The affection was not more frequent then than when it first began. The man looked sickly, as if he had had ague, but still more as if he had suffered from a hot climate, and it appeared that he had been in the East and West Indies, and that he had had fever both at Bombay and Batavia. He had suffered from dysentery, and when he was in the hospital he had diarrhœa. I do not doubt that this was the effect of malaria—that his hemiplegia was a form of ague. I will not quarrel about words, you might say it was not ague, because unattended by shivering, fever, or sweating; but I have no doubt it was as much the effect of malaria as ague is: it was merely a variety of the same affection of the system. Supposing this to be the case, and having witnessed a paroxysm myself, I now gave him the sulphate of quinine, and, as the disease was of long standing, I began with a good quantity,—five grains every six hours; this medicine very soon put a stop to the complaint, but not till I had increased the dose to ten grains every six hours, so that he took forty grains in the twenty-four hours. This is the dose that is often required in quartan ague, and the present was a worse form of the disease than quartan, because it occurred on the third or fourth day, and the longer the interval between the attacks, the greater is the difficulty of curing the affection, which may be considered as so much the more of a chronic character. It is not a matter of wonder that that large quantity was required. He continued in the hospital from his first admission on the 13th of October till the 23d of December,—rather more than three months, without any other attack whatever, and his health became greatly improved. It is wrong to suppose that malaria does nothing more than produce these particular forms of intermittent disease; it poisons the whole body, and many persons are destroyed by it who never had ague at all, so deadly is the poison. His health, however, regularly improved under the quinine; he became strong, his countenance was better, and altogether he found that he had received very great benefit from it. However, on the 28th of the same month, five days after his presentation, he came to me, saying that he had had a slight attack, a very slight one, that morning, but still it was an attack, and it occurred rather later than usual, some little time after 11 o'clock. When I saw him, at about half-past one o'clock, it was then nearly gone off. I increased the quantity of sulphate of quinine to fifteen grains every six hours, and if that be not sufficient I shall give him more, as he is to come to me from time to time. I had a person in the hospital who was not cured of ague with less than a scruple every six hours, and therefore I shall not be surprised if that quantity be required in the case of this man; but I have no doubt that eventually he will be perfectly cured, though he may need very large doses.

This is a very interesting case, proving that paralysis is not *necessarily* an *organic* affection; that hemiplegia does not necessarily arise from effusion, or from compression of any kind, at least of an organic nature. If any compression do occur in this man, it can only be during the fit, for at other times he is perfectly well; it is entirely, I presume, an affair of *function*, induced by a particular poison. I have at this moment in private practice a very curious case, in which disease has arisen from malaria; it has occurred in a young gentleman about eleven years of age, who lives by the side of the Thames. He had diarrhœa at school, which was allowed to run on; he was, however, taken home, and treated very properly by the gentleman who attended the family, by leeches to the abdomen, and I believe a blister, and all went on very well. He had tenderness just on one side of the umbilicus; he was, however, seized all at once, at a certain hour of the evening, with violent irritation, severe itching, tingling, and redness at the leech-bites, some feverishness, just at the very part where all the leeches had been applied, and every leech-bite became red and swollen. His sufferings were extreme, but after lasting for a certain time, all these symptoms went away. At the same hour the following evening the same thing occurred, the leech-bites became swelled and hot, and he fell into a state of general excitement, from, as it would appear, the itching and tingling. The medical gentleman immediately saw him, and thought the attack was of an aguish character, and, as the family lived in a low spot by the side of the Thames, he gave this lad twenty grains of sulphate of quinine, in divided doses, before the time of the next expected paroxysm. The attack came on the next evening, but at a later period than usual, showing that the remedy had produced an impression. It is common for the remedy not to stop the disease at once, but to cause the fits to be postponed. I, however, was sent for, and I told the family that I was quite satisfied that the youth was going on right; that the quinine was the only remedy, and that it must be persevered in at the same doses. The fits were very distressing, indeed to the family alarming, and we both agreed that it was better to go on with twenty grains in the twenty-four hours. The next day the paroxysms appeared later and more slightly, and then came on once in two or three days, and still more slightly; he presently became perfectly well. At the end of a month he went out of doors, and was exposed to cold, and from his extreme anxiety to regain the time he had lost from school, for he was a fine boy, a paroxysm came on again, but rather mildly; the medicine was again had recourse to, and the immediate effect was a postponement and alleviation of the next paroxysm, and I have no doubt that if he continue to take the remedy for some weeks, he will not have a relapse. These remedies will not cure the disease unless you give them for some time after the disease has appeared to cease. Sometimes it is necessary to give them for many weeks; sometimes it is necessary to do more than this—to remove the patient from the spot. Just as in syphilis, if a person get cured, and return to the same quarters, the mercury he has taken will, of course, not prevent him from again catching the disease; so a person may be cured of ague, but if he continue to live in the same unhealthy quarters, of course the poison may operate afresh upon him; and as in syphilis mercury must be taken for some time after the symptoms have all disappeared, so must quinine *after* ague."

We have seen many cases where paralysis was evidently not the effect of organic disease, but of malaria. One, which we are not at liberty to mention by name, but which we lately saw with Dr. Paris, (who will confirm the truth of this statement) is still more remarkable than the case recorded by Dr. Elliotson. A young gentleman, the son of one of the most PUBLIC CHARACTERS in England, travelled, more enthusiastically than wisely, through Italy during the Summer of 1830. The consequence was, a malaria fever, after which he lost the power of *all* the voluntary muscles of the body, from head to foot! In this deplorable condition he was conveyed from Venice to London, *like a corpse in perfect health.* All the functions went on regularly—intellectual and organic—but all animal power of motion and locomotion was lost. This general paralysis continued for some weeks after his arrival here; and Dr. Paris very judiciously abstained from violent remedies, such as strychnine, &c. trusting to the recuperative powers of Nature. Motion gradually returned —first in the upper, and afterwards in the lower extremities—and we believe he is now walking about, and enjoying good health. Such accidents, though seldom on so remarkable a scale, are every year happening in Italy and other malarious countries, though they are very imperfectly known to the profession of Great Britain.*

### III. ABSCESS OF THE OS FEMORIS—INFLAMMATION OF THE LEG—DEATH.

John Gilchrist, aged 45, was brought

* Vide Dr. Johnson on "Change of Air," 1831.

to St. Thomas's Hospital at 2, p. m. November 23d, 1830, with dislocation of the head of the humerus into the axilla, occasioned only a few minutes before, in falling off the outside of a stage coach. The symptoms of this injury were exceedingly well exhibited, and, from the short period that had elapsed since its occurrence, no difficulty was experienced in replacing the bone. This was done by making extension at an angle from the trunk; and bending the arm over the knee of the operator, on which it was placed, the head of the bone almost instantly slipped into the glenoid cavity. The patient also fell to the ground on the left thigh, in which chronic disease of the bone with sinuses and discharge, and anchylosis of the knee-joint, consequent on the diseased bone, existed. Some contusion was produced at the lower part of the thigh, and he suffered great pain from this part. A roller was applied around the arm and trunk to secure the former to the side, and when he was placed in bed, his thigh was supported on a pillow, and kept wet with an evaporating lotion.

24th. Has slept but little on account of pain in his thigh. The shoulder has been quite easy. 27th. No sleep has been obtained, in consequence of frequent and severe twitchings of the thigh, which have continued by day as well as night: there is no appearance in the limb to account for this. Shoulder perfectly easy. Dec. 1st. Severe pain has been felt in the thigh during the last three days, but no inflammation of the surface or deeper parts can be detected. 3d. Leeches have been applied with great relief of pain.

13th. An atonic kind of erysipelas has attacked the leg, and gangrene is threatened on the foot and ankle.—Great swelling has taken place from the knee to the tarsus, arising from effusion of fluid into the cellular tissue; the integuments are of a faint red hue, and the cuticle is desquamating in different parts, though vesicles have not formed. The surface is tense and shining, but less so since an incision of two inches in length has been made on the outer side of the tibia in the middle of the leg, from which serum is now flowing abundantly—pulse is very feeble, 100—skin cold and clammy—countenance pale and sunken—hiccup during the last 24 hours, frequent but not powerful—dryness of the tongue and mouth with thirst. A little wine was ordered for him, and as it was suspected, from the tense state of the integument, that matter was forming beneath the cellular tissue, three smaller incisions were made about the middle of the leg, on different sides. The skin was relieved of tension thereby, and some serous fluid was discharged, but no pus.

15th. The cuticle is desquamating from the whole leg, but the tension and redness are much abated. Patient continues equally low—hiccup very frequent. The edges of the incisions seem disposed to slough.

20th. Since our last report the patient's strength has progressively declined—pulse is now 110, excessively feeble—the incisions are surrounded by sloughs to a considerable extent, and a large gangrenous patch has formed upon the instep. There is considerable discharge of pus from the incisions, and the leg is as much enlarged, with as great redness of the integuments as before; he however takes freely of nourishment and wine; poultices of linseed meal are applied with solution of chloride of lime in direct contact with the sloughing parts.

26th. His constitution appears to have held out surprisingly, he being in the same feeble condition as at the time of the last report; the sloughs appear to be spreading still further around the incisions, which afford a free discharge.

30th. He is somewhat improved. His pulse 100, not so feeble as it was lately; his bowels are regular, and he has some relish for food, which he takes in larger quantities, and of the most nourishing kind. The leg is likewise a little better in its condition; it is less swollen and red; there is a plentiful discharge from the incision, the sloughs from which have not yet separated, but their limits are defined.

Jan. 2d, 1831. Patient is in much the same condition, but his pulse has acquired more power; he rests well, and makes no complaint of debility or pain.

4th. The sloughs have entirely separated from the wounds, and a clean and smooth granulating surface is exposed in the different parts of the leg, such as would be expected in a state of the system where deficient power existed like the present; on the dorsum of the foot the extensor tendons are exposed, and there exists an ulcerated surface larger than the palm of the hand, left from the separation of the gangrenous patch noticed on the 20th ultimo. The tibialis anticus muscle is also laid bare about its middle, the general swelling is greatly reduced, as well as the discolouration. A profuse discharge of pus yet exists, which in some situations burrows beneath the skin in excavations formed by the sloughing. There is extreme tenderness upon pressure near these wounds, when made to evacuate the matter. Pulse 100, not feeble, quite regular. Tongue clean and moist. Takes nourishment liberally, and a manifest improvement is observable in his looks.

9th. A very profuse discharge is kept up, and the granulating surface of the wound is still smooth and polished, shewing very little disposition to heal. Pulse 110, more feeble; the appetite not so good, and emaciation has become perceptible.

14th. The discharge of matter has very much increased, and it now burrows downwards from each sore, and there would collect, if it were not carefully expressed every time the poultices are removed.

18th. No diminution in the quantity of pus, which has become excessively fetid.

26th. A fresh opening has been made in the thigh near the groin, where matter had collected, and whence an immense quantity of pus is now discharged. The discharge from the cavities in the leg, which has lately shrunk a good deal, is no less profuse. His appetite has failed him, and he is supported only by the wine and brandy he takes in gruel, arrow-root, &c. The pulse has become very feeble, 100; the tongue is clean; he does not complain of pain, but he has night-sweats, and becomes weaker daily, from the profuse discharge of matter.

30th. Has been progressively sinking, and died to-day about 5 o'clock, p. m.

*Post-mortem Examination.*

The whole limb was pale, shrunk, soft and œdematous—the knee-joint was firmly anchylosed, and there was now detected, for the first time, a complete separation of the os femoris into two portions. This was the consequence of an abscess which occupied the bone at the junction of its middle and lower thirds. The sac of the abscess was well defined, and seemed to be formed by the periosteum. At each extremity of the bone a portion of its end had become necrosed, and was undergoing at this time the process of exfoliation. The granulations which had arisen from the separated ends being interposed between the dead and living parts, they were in considerable quantity. Some pus, with fragments of dead bone, was contained in the cavity of the abscess. The whole of the bone was much enlarged in size, evidently shewing that the disease had existed a great length of time. The anchylosis, however, seemed to have been more recent, as the connexion was only of the ligamentary kind. Numerous sinuses were found throughout the limb, which communicated with the external sores—the muscles had become thin and pale from their long inaction, and the whole of the cellular membrane was thickened from effusion of lymph and serum. No other part of the body was allowed to be examined.

### IV. DISEASE OF THE SPINE.

William Danyer, ætat. 29, a miller, was admitted into St. Thomas's Hospital, July 8th, 1830. He stated that he had taken cold four weeks ago, after which he had been attacked with pain of the back between the shoulders, and also sensation of stiffness and weakness in the knees and ankles. He says that the pain of his back sometimes shifts into his sides; and also describes his knees and ankles as being much easier when warm in bed; in other respects his health is pretty good, he eats well, sleeps tolerably, pulse 80 and weak. Having no paralysis or deficient sensibility at this time, and referring his ailments so entirely to his joints, coming on after cold, his case was considered to be simple rheumatism, he was therefore ordered the warm-bath every evening. Tinct. guaiac. ammon. ʒiss. 6tis horis, and sulph. quin. gr. ij. 6tis horis. 17th. No amendment; has been taking the medicines regularly as above prescribed, together with the liniment; sinapisms to the knees. He is ordered to increase the dose of tinct. guaiac. amm. to ʒij. and the sulph quinin. to gr. iv. 6tis horis.

Aug. 4th. No alteration taking place under this treatment he was put upon the carb. of iron, which he took in doses of ʒij. increased to ʒiij. 6tis horis—and a moxa was applied to the loins. 18th. The stiffness of his knees and ankles has increased since last report; in other respects much the same. He was ordered vini colchic. ʒss. ter in die, which was continued for some time without improvement. Sept. 10th. He has now symptoms which leave no doubt that his ailments are to be referred to something more than rheumatism; he feels numbness in his lower extremities, which often causes him to trip whilst walking, and he has also occasional twitchings of the muscles. On examination of the spine there is not any irregularity perceptible, but he complains much of the pain, referred rather higher than the loins; a plan of treatment was therefore adopted, more particularly directed to this situation: he was cupped repeatedly, and had blisters applied over the seat of pain—the only medicine which he took being aperients. Notwithstanding a very active application of these means he became much worse; the numbness increased to total insensibility; the spasms of the muscles became much more frequent and severe; he lost all muscular power of the lower extremities; his urine became retained, and he passed his fæces involuntarily; the loss of feeling involved the coverings of the lower part of the abdomen, as well as the lower limbs. He was now confined entirely to his bed, being unable to make the least voluntary use of his muscles. He continued during the following three weeks without any amendment; he was quite bed-ridden, and all the symptoms unalleviated, when, about the 11th of October, he was attacked with acute peritonitis, which speedily terminated in death.

*Autopsy.* On examination of the spine, a considerable quantity of curdy scrofulous matter was found deposited beneath the anterior spinal ligament of the three lowermost dorsal vertebræ. The arches and spinous processes being removed, it was found that the vertebral canal was much encroached upon by deposit of the same kind, beneath the posterior ligament. The extent of the disease in this situation was the same as in front, and occupied the same vertebræ. The quantity of matter formed was sufficient to press on the spinal cord, and thus induce the paralysis of the parts below. The bony parts of the vertebræ were perfectly sound, but the intervertebral substance was exceedingly soft between the 10th and 11th, as also between the 11th and 12th dorsal vertebræ; and it was in this situation that the deposit was in greatest quantity. The matter was not however isolated in these two situations; a probe might be passed with readiness under the ligament from the anterior to the posterior collections round the entire vertebræ and intervertebral substance: the ligament was three times its natural thickness, and cut almost like tendon. This case will no doubt he considered interesting, as demonstrating the cause of paralysis to depend upon scrofulous deposit, without diseased bone, and likewise occurring in an adult in whom no other marks of scrofula were evident.

### V. PARALYSIS FROM FRACTURED VERTEBRA.

William Dunlop, a stout labouring man, about thirty years of age, was admitted into St. Thomas's Hospital March 25th, 1830, with complete paralysis of the lower half of the body. He stated that, eight weeks previously, he had fallen into the water, and that a large piece of wood struck him on the back at the same time. When taken up, he had paralysis of the lower limbs; he was taken home, where he remained for two months, passing his urine and fæces involuntarily. At length, from remaining on his back so long, the integuments over the sacrum sloughed, and he was then brought to the hospital. When admitted he was perfectly insensible, and had lost all muscular power below the seat of injury—the *urine* and fæces passed involuntarily, nor did he ever require the introduction of a catheter, the urine seeming to flow away as fast as secreted: the lower extremities were œdematous, and a slough existed on the sacrum—he did not complain of his back, indeed he did not make any complaint, bearing his sufferings with great fortitude—the only means adopted were, attention to the state of the wound on his back, and affording him what nourishment he was able to take, together with the occasional use of enemata. At length, from the distention of the limbs with fluid, and the pressure on the bed, the integuments covering both heels sloughed; and, after continuing in this miserable state of existence until the 10th of September, he died worn out by the discharge from his heels and back.

On examination of the body after death, the spine was found bent to a considerable angle towards the right side. On more particular examination, it was found that the arches of the 12th dorsal vertebra had been fractured, and also that a portion of the front part of the body of the first dorsal vertebra was split off, and driven downwards below the correspondent intervertebral substance—the vertebra itself was driven forwards two-thirds of an inch, and the intervertebral substance had nearly disappeared—the process of reparation had gone on to a considerable extent; the broken arch had re-united, but it was in an irregular manner, the spinous processes of the two vertebra being farther separated than natural, and a considerable angle being made behind—the portion of the body broken off had also become anchylosed in its new situation—the sheath of the spinal cord was intimately united to the injured bone; and several of the nerves, from the cauda equina, were involved in the same adhesive process—the rest of the cord was embraced by a deposit of bone in the arachnoid covering, forming a complete osseous ring—in consequence of the displacement of the vertebra, the size of the canal was much contracted and very irregular in shape—the viscera of the thorax, abdomen, and pelvis healthy.

### VI. EMPYEMA.

Joseph Jacobs, aged 21, was admitted into St. Thomas's Hospital Nov. 18th, 1830. Says that he has had a cough during the last twelve months, and has become much thinner—has never spit any blood. About a fortnight ago he was attacked with pain in the chest and head, and shivering—the pain of the chest confined to the left side, and increased on inspiration. Pressure between the ribs gives him pain. Two days after this attack, he began to spit a thick white matter, which continues to the present time. On percussion, the chest sounds very dull anteriorly and posteriorly—the respiration is scarcely audible at any part of the left side. Pectoriloquy near axilla—he is unable to lie on the right side without coughing and great increase of pain—has considerable perspiration at night—his bowels relaxed—pulse 128—tongue white—is emaciated, and has a hectic flush on his cheek. It appears that he has led a very intemperate life, and lately has been exposed to cold and wet. He has a sore at the present time on his penis. He was ordered empl. cantharidis pectori—opii, gr. ij. o. n., acid. sulph. dilut. ♏xx. 6tis horis, ℔ij. strong beef-tea daily, arrow-root and sago. 24th. His bowels are greatly purged; in other respects the same. ℞. Infusi catechu. comp. ℥jss. t. d. 27th. The medicine changed to t. opii, gr. vj. t. d. He went on varying somewhat in the intensity of his symptoms, but without any permanent improvement in his health; his strength supported by food of a nourishing kind, until his bowels became again much purged, the colliquative sweats continued, and he gradually sunk.

On examination, it was found that the left pleural sac was completely filled with puriform matter, the pleura costalis much thickened, and large flakes of lymph were hanging from its surface; the ribs had in many places become carious where in immediate contact with the diseased pleura; the lung was closely compressed to the back of the thorax, to which it was adherent; some small cavities existed in its upper part, and numerous spots of tuberculous matter were deposited through its structure. On the right side, the lung was found adherent through its whole extent to the pleura costalis; the adhesions very close and firm—the left testicle was enlarged from scrofulous disease, which extended through the entire of the vas deferens, and involved the vesicula seminalis of the same side. This body was rendered solid, and as large as a hen's egg—two or three tumours of the same kind were formed in the prostate gland—the right vesicula and vas deferens were healthy, but their common duct was impervious where passing through the prostate.

### VII. DISEASE OF THE HEART.

William Herbert, æt. 27, was admitted into St. Thomas's Hospital Jan. 20th, 1831. He states that a little more than three months ago, he was attacked with pain of the left side of the chest, soon followed by cough, and attended with difficulty of breathing—with palpitation of the heart—has some pain in the abdomen, especially at the right hypochondrium, increased on pressure; there is tension of the epigastric region; he says that the abdomen has become swelled within the last fortnight; there is now very distinct fluctuation, and, moreover, the whole body is anasarcous—he cannot lie on either side, and requires to have his shoulders raised—the stethoscope indicates respiration natural over the superior part of the chest on both sides, anteriorly as well as posteriorly, and also at the inferior part of the left side, but inaudible on the right side, at which part the sound is very dull on percussion—the impulse of the left ventricle very strong, with a grating bruit after the ventricular contraction—urine scanty—pulse 120, sharp.

He was bled to twelve ounces, a blister applied to each side of the chest, and was ordered diuretic medicines, with v. grs. hydrargyri c. cretâ every six hours; the bleeding was repeated on the 22d, and his medicines continued. Blisters were applied successively to his back and sides, with p. jalapæ comp. ℈ij. Under this treatment he improved considerably; his bowels becoming affected, the hyd. c. cretâ was omitted, but he continued to take the diuretics. He was cupped on the 16th Feb. with relief to his symptoms, and became so much better that he was able to sit up. On the 17th, however, he complained of increased difficulty of breathing, and, on the following morning, fell back in a fit of syncope, in which he died.

About three pints of fluid were found effused in the right pleural sac—the heart was much enlarged in its entire extent—the right auricle and ventricle were enlarged, and the auricle more fleshy than natural—the left ventricle greatly dilated, at the same time that its parietes were of increased thickness; with the exception of a very small fleshy deposit on the mitral valve, this opening was quite healthy; but the semilunar valves of the aorta found to be much altered in their state, and that situated most posteriorly had been either torn by the blood forced through the mouth of the vesssel, or, which is the most probable opinion, it had become ulcerated. One half of the valve had disappeared, with the exception of a mere shred of its investing membrane; the other valves were thickened and less mobile than natural—there were some marks of previous inflammation in the abdominal cavity.

DELTA.

### VIII. TUMOURS AT FIRST INNOCENT BECOMING MALIGNANT.

No subject can be more interesting, or practically important to the surgeon than that which the above designation embraces. The three cases subjoined will supply ample matter for consideration upon this topic, and we trust its importance will plead an excuse for their length and the details we shall enter into.

CASE 1.—*Tumour near the Angle of the Jaw.* William Logan, æt. 49, from Maidstone, admitted Dec. 2d, 1830, under Mr. Green. He has a firm and solid tumour situated on the right side of the face and neck, covering the angle of the jaw and parotid gland, and the size of a large orange. The dimensions of it are very easily traced, the part being a remarkably distinct growth. It is nearly circular in shape, and apparently projects altogether outwards from its connexion with the surrounding parts; it is moveable to some extent, and seems to have no firm or deep attachment; some portion of it is traceable on the inner side of the angle of the bone, where the patient informs us any enlargement was first observed as far back as twelve years ago. At that distance of time a tumour little larger than a walnut existed just below the angle, and could be moved about with facility in every direction. The enlargement extends in front nearly to the commissure of the lips, or about two inches from the symphysis of the chin, here terminating by a thick and abrupt border; above, it reaches to the malar arch, at which point its strongest superficial attachment exists, adhering pretty firmly to the fascia of the masseter muscle by a process from the body of the tumour which becomes gradually thinner as it ascends; behind, it is defined by a thick edge above an inch from the angle of the maxilla, approaching between the latter point and the preceding close to the meatus auditorius externus, and thus displacing the lobe of the ear; below, it descends upon the neck in a thick mass about an inch and a half from the horizontal ramus of the maxillary bone. Its surface is somewhat rounded, the most prominent part being in the centre, and communicates a firm and lobulated sensation to the fingers. There is one small lobule, or portion, distinct from the bulk of the tumour, at its upper and anterior part. The progress of the enlargement has been very slow, and unattended with pain of any kind, or other sensation, so that from aught he feels he would be quite unaware of its existence. During the last three years he has noticed very little increase in its bulk. No local or general irritation is produced by it; his hearing on that side is unaffected; the parotid gland, or its duct, does not seem influenced by pressure from it, as the saliva passes freely into the mouth on that side through the duct of Steno. There is no impediment to mastication or deglutition; no difficulty of breathing, or cough (from the latter he has suffered at times, and attributed it to the swelling irritating the throat from its size;) and regarding his general health, he has no complaint to make.

It was looked upon as a common sarcomatous tumour, to which opinion the history given would naturally lead, and not of a malignant character, but yet not safe to be allowed to remain.

Dec. 10. The tumour was to-day removed. An incision was made through the skin, commencing at its anterior prominent border on the cheek and terminating behind at a short distance beyond the lobe of the ear, being carried over the body of the tumour in a somewhat semilunar form. The superficial connexions of the cyst were slight, and admitted of being broken through by the handle of the scalpel. Near to the malar arch they were stronger, and required the use of the knife. The cyst was found attached to the parotid gland so intimately that the condensed cellular tissue of the latter was with difficulty distinguishable from it; some of the lobules of the gland were visible at its lower part, but were distinct from the tumour, which was dissected from this situation without any lesion of the parotid. Another process of the tumour passed deeply downwards close to the external carotid artery. The bulk of the tumour was now cut off from this portion, which it was necessary to take away with great care. At this time the artery could not be felt by the finger, when placed directly over it in the wound. After the removal of this part, the external carotid was completely exposed for the space of a quarter of an inch, and its pulsations were very manifest. Two small arteries were tied during the operation, and two more before the man was removed from the theatre, but they were not so large as might have been expected from the size and long-continuance of the tumour. Fearful that hæmorrhage might occur, Mr. Green deferred the dressing of the wound till the patient should become warm in bed. A dossil of lint was therefore simply laid over the incision. After the patient had been in bed about an hour the carotid artery beat so forcibly that the lint was moved by each pulsation, and the motion was visible at a distance; bleeding shortly took place, and rather a large quantity of blood was lost in a short time. With some trouble and difficulty four bleeding vessels were secured in different parts of the wound, one more deeply than the rest, near the carotid trunk. The integuments, now that the tumour was extirpated, were flaccid and loose, and consequently brought together with facility by strapping. A damp linen cloth was then laid over the part.

A section of the tumour was made, and it consisted of several small cysts, closely pressing against each other, containing a thick glairy fluid of a yellow colour, intermixed with specks of dark coagulated blood, and having dense and strong fibrous partitions. The external cyst of the tumour was rather thin, and closely enveloping this internal structure. In the centre of the mass there was a whitish and hardened portion, approaching to a cartilaginous texture. The morbid growth was considered malignant, but in an incipient state.

Dec. 10th. *Vespere.* No return of bleeding—patient easy—pulse 90, of natural force—skin warm and moist.

11th. Had a few hours sleep last night—no uneasiness felt in the wound—some pain of head—pulse 100, full, but not hard—tongue rather dry, and yellow in the centre; the latter appearance was noticed before the operation—has some thirst—skin rather hot, though moist—bowels were open yesterday morning, but not since—Mr. Green directed ℥x. of blood to be taken from his arm. Cal. gr. v. hâc nocte. Mist. sennæ co. ℥ij cras mane.

*Vespere.* Relieved by the bleeding—head free from pain—pulse 96, less full, and smaller—skin moist—no stool.

12th. Passed a quiet night with a few hours sleep—is in no pain—pulse 96, small and compressible—tongue cleaner—bowels opened. Some swelling is observable around the dressings, more particularly downwards on the neck below the wound, but it is not productive of uneasiness.

*Vespere.* Quite easy—the tumefaction has rather increased upwards on the face.

13th. Feels comfortable after another easy night. Swelling under the chin much subsided, but more conspicuous on the face—pulse 88—bowels open. The straps were removed for the first time, and the edges of the wound found in close contact—there is a slight discharge of pus from one part—the swelling that has taken place feels firm and has rendered the skin, which was previously loose, in some degree tense. There is, however, no redness, or pain on pressure—straps replaced at a slight distance apart, with simple dressing and bread poultice.

13th. Swelling quite subsided, and the edges of the incision are closely approximated—pulse 80, perfectly soft—bowels open.

15th. Expresses himself free from ailment, having passed a good night.

21st. No unfavourable symptom has presented itself. He has rested well, and taken the food allowed him. The wound has united throughout, and two ligatures only are remaining. Some enlargement is perceptible below the jaw, firm and hard, a little distance below the inferior extremity of the incision.

The ligatures had all separated by the 24th, and on the 6th January, 1831, he quitted the hospital. On the 2d he experienced slight pain in the neck, in the part mentioned as being enlarged, but this had ceased on the following day, and no increase of swelling had taken place.

Case 2. *Tumour on the Arm.*

Henry Gooding, æt. 24, a bricklayer by trade, applied at St. Thomas's Hospital, December 23, 1830, on account of a large swelling situated on the upper arm, and was admitted a patient under Mr. Green. This swelling has unequivocal appearance of malignity, and it occupies principally the outer, back, and inner part of the middle third of the arm. Commencing a little below the rotundity of the shoulder, it descends between three and four inches towards the elbow, and is in this situation harder to the touch, more prominent, and also more circumscribed than at any other point; here also we are informed the disease originated two years ago. From the outer side of the arm the tumour takes its course in an upward direction backwards and inwards to the inferior boundary of the axilla; it is throughout this extent softer, more diffused and undefined, and the integuments are less tense. The whole enlargement imparts the sensation of a firm and ponderous tumour upon being handled. In some parts it is hard and resisting, whilst in others there is an elastic feel perceptible, similar to that of fluctuation. At the most prominent part the skin possesses a slight red tint, limited to a very small space; and posteriorly some enlarged veins are observed tortuous near the surface. The cephalic vein has likewise become of a larger size than natural in the neighbourhood of the disease. The tumour is immoveable from its attachments, which, to appearance are in the cellular and muscular substance of the arm, the bone not being implicated. This opinion, it will be seen, was verified by the dissection. Till within the last six months the patient has lived in Huntingdonshire, but during this period he has been employed in London, in Long Acre, the motions of the limb being impaired, but not to such a degree as to prevent him from working up to the present time; indeed, considering the magnitude of the tumour, he can use the arm with great freedom. Within these six months too, the disease has acquired its principal growth; and latterly its development has been the most rapid, the pain, which he describes as sharp and darting through the whole substance, increasing in the same ratio with its bulk. There is no glandular enlargement to be felt in the axilla, but considerable heat exists upon the surface of the swelling. The general health of the patient, as his appearance would testify, has been good, and his parents were, from his account, of sound constitution. His pulse is under 80, and of natural character. The tongue moist and perfectly clean.

Dec. 28th. Emplast. ammoniaci, c. hydr. applied to the arm.

Jan. 7th, 1831. The patient says he has found the pain increase day after day since his admission. In the tumour itself there is little alteration apparent; the superficial veins on its surface are perhaps seen more conspicuous, together with a slight augmentation of redness and heat. After some demur on the part of the patient as to losing his limb, which he little expected, and was consequently taken by surprize at the proposal, amputation was this day performed at the shoulder-joint by Mr. Green. Mr. Tyrrell effectually compressed the subclavian artery above the clavicle, where it passes over the first rib, with a padded key, and the radial artery could not be felt at the wrist. The operator commenced by making a semicircular incision with a large double-edged scalpel from without inwards to the axilla, passing from about an inch and a half above the posterior fold of this cavity and a little below the prominence of the deltoid muscle upon the arm, the concavity of the incision looking upwards, to a little distance upon the parietes of the chest. He recommends this incision to be made freely, as less difficulty will be experienced in passing the knife between the head of the humerus and glenoid cavity. The deltoid was then separated from the humerus by the catlin, to form the anterior or upper flap. The head of the bone being now carried outwards by the assistant, the instrument was passed between it and the glenoid cavity of the scapula, and the capsule divided. Disarticulation thus effected, the operation was concluded by cutting through the soft parts on the inner side of the limb obliquely downwards and inwards, and the lower flap, smaller than the upper and consisting chiefly of integument, was made in this situation. The axillary trunk, the subscapular, and the posterior circumflex branch were tied with silk ligatures. Bleeding from two other vessels of considerable calibre was arrested by *torsion*, after the manner of M. Amussat, and with an instrument made by Laundy for this purpose. The instrument is very simple, having two blades like those of common arterial forceps, roughened at their extremities, and a slider which prevents their separation when the artery is taken hold of and twisted. About eight or ten ozs. of blood might be said to have been lost, and the man was for some little time in a state approaching to syncope, the pulse being very feeble and the surface chilly; he was however, revived by two glasses of wine, and lowering the chair on which he was sitting so as to bring the head and trunk nearly in a horizontal line. The flaps were then approximated by strapping, which was applied first at both extremities of the wound, and lastly over the centre. A long and broad piece of the plaster was carried from below the breast over the shoulder near the acromion, and some way down the back, in order to depress the point of the shoulder; and the surfaces of the stump in contact were supported by a compress of lint placed over their line, and this confined by a strip of plaster. Our patient went through the operation with great firmness and self-possession; for a short time after he was removed to bed he was rather low, and felt cold and thirsty. Hot flannels were applied to the feet, and the body in general well covered. This state of depression soon went off without the exhibition of a stimulus.

Jan. 7th. Nine o'clock, p.m. Patient has had some little sleep, but the stump has been rather uneasy from smarting, and it has shewn a disposition to starting. He appears somewhat restless, and has continued thirsty; has nausea, and at one time retched, but did not throw any thing from the stomach. Pulse 88, rather full—skin temperate—mouth dry, but the thirst is allayed for a time by barley-water—no pain of head, or any internal organ. Has made water easily. A free sanguineous discharge from the stump, principally of venous blood. Mr. Green considered that if rest were not obtained in the night, of which he saw no probability at present, the patient would be found in the morning in a feverish state, and it was evident there was now no want of power or liability to febrile action in the system. In fact, he said, excessive re-action was very likely to succeed to the depression which ensued after the operation, in a vigorous constitution like the present. He therefore directed 30 minims of tinctura opii to be administered immediately, and if the pulse should become hard, blood to be taken from the arm.

8th. About three hours after he took the opiate sleep came on and continued for some time. He lies quiet, and without pain—feels numbness in the stump, but this is not restless—pulse 88, compressible, and not fuller than natural—skin naturally warm and moist—thirst and dryness of mouth very much diminished—tongue not white or dry, indented at the margin by the teeth. Urine has passed freely, but no evacuation has taken place from the bowels; as he had no unpleasant symptom, however, arising from the want of alvine excretion, Mr. Green thought it adviseable not to disturb him by a purgative. Takes barley-water, gruel, &c.

9th. Slept for many hours last night without an opiate, and feels quite comfortable. Numbness continues in the stump, but there is no pain, or aching, from the dressings, which have retained their position. Pulse 88, as yesterday—skin cool—tongue moist and clean, still marked by pressure against the teeth. Bowels are not yet open.

10th. Passed another easy night, but was occasionally disturbed by the coughing of a patient in an opposite bed. He is in no pain—skin is rather warmer than yesterday—pulse 86, and in some degree full. Has had no motion, but is ordered a dose of ol. ricini. Partakes of beef-tea, &c.

11th. The oil opened the bowels freely last night. Pulse when this morning felt by the dresser was at 72 —at Mr. Green's visit it was 88. Patient is free from thirst, or pain — his rest was undisturbed, and the skin is cool. Dressings quite dry, and firm. Allowed bread pudding with an egg, and beef-tea.

12th. Pulse 80—tongue moist, continues indented. Dressings easy, and were not interfered with. The bowels have not acted since the night of the 10th.

13th. The straps were all removed to day, and it was found that owing to the oozing of venous blood for some time after the operation they had become displaced below the wound, and the lower flap being thus ill supported was separated from the upper for nearly two inches. The surface was, nevertheless, quite healthy, and there was no discharge from it. He felt rather uncomfortable with these dressings, but when fresh ones were applied from below upwards, which brought the flaps much nearer in contact, he was easy—rests well—bowels not open; he has therefore taken house medicine—pulse 80. To have fish for dinner, and more generous diet.

14th. As no action of the bowels was produced by the first dose of medicine, a second was given last night, and three motions have been obtained this morning. The dressings seem to have slipped a little from the lower part of the stump—mutton-chop, and half a pint of porter.

15th. Was again dressed, and the granulations of the wound are perfectly healthy.

17th. Some coagulated blood, which had collected in the stump rather deeply after the venous oozing, was removed, and the straps of plaster placed in a more oblique direction, lint being interposed between them and the surface of the axilla below the inferior flap to support the latter.

20th. The flaps are retained in apposition, and the granulations have united over the part whence the coagulum was taken out. There is every prospect of a speedy recovery, the patient continuing without any untoward symptom. He now takes a pint of porter in the day, with mutton-chop.

28th. Patient is quite comfortable, and free from pain. Wound granulating rather luxuriantly, and is in consequence touched with argent. nitras.

Feb. 4th. One ligature has separated and the wound is contracted to a very narrow and healthy granulating surface.

11th. Has often sat up to have his bed made — is quite well in general health, and the healing of the stump has proceeded uninterruptedly.

17th. The last ligature came away to day, and a small granulating line only remains to be cicatrized. He continues out of bed nearly the entire day, and makes complaint of but slight debility. A glass of wine is substituted for the porter, and mutton-chops are allowed daily.

*Dissection of the Arm.*

When the arm was dissected, the following state of parts was found. The biceps muscle was of very large size, and was extended laterally so as to occupy the entire anterior portion of the arm; its substance was very little, if at all, thinner than natural. On the outer side of this muscle, just below the deltoid insertion, the tumour presented itself of a white colour beneath the integuments, and some little distance lower down behind the brachialis anticus, which was so much protruded forwards by it that there was an interval of two inches between its outer border and the supinator radii longus, which space was filled by the prominent part of the enlargement. The brachialis fibres were expanded from side to side, and consequently thinned in proportion. The tumour passed downwards rather to the outer side of the humerus to within three inches of the external condyle, where it was smaller in bulk, and its termination distinct. Its course from the outer side of the arm, from the point first mentioned, was upwards and backwards beneath, or rather anterior to, the triceps extensor (which was thereby put upon the stretch,) then round the inner side of the limb, being close to the bone and separating the two shorter heads of the triceps near their origin. It terminated in the axilla by a rather narrow process, which extended as far upwards as the tendinous insertion of the latissimus dorsi, and therefore about an inch higher than that of the teres major, which is described as the lowermost boundary of the cavity of the axilla. Close to this portion of the tumour the catlin passed in the amputation, as it was seen without dissection on the wound of the amputated limb. There was slight displacement forwards of the brachial artery and median nerve. The whole tumour consisted of four separate lobes connected near to each other, some being of harder texture than others. The entire external surface was white and solid. No true capsule could be said to exist, the mass being enveloped in condensed cellular tissue, which being torn off, displayed the tumour itself, having a surface compared in appearance to that of the convolutions of the cerebrum. It was attached to the periosteum simply by cellular adhesions broken through with ease. Upon section its internal structure was found like that of the pancreas, and possessed very much the characters of the pancreatic sarcoma described by Mr. Abernethy, consisting of larger and smaller lobes closely connected together. In one part the texture very much resembled scirrhus, both to the eye and touch; and, in another, there was a small excavation of a suspicious character, the surrounding substance being softened, and some black coagulated blood effused around the opening. The spinal nerve was imbedded in the centre of the most prominent lobe.

Mr. Green remarked in a clinical lecture on the 14th January, of which the latter case was the subject, that it appeared to him and his colleagues nearly all the characters of fungoid disease were here presented, yet the enlargement was unlike almost every other description of tumour, being hard in some parts, and in others soft, and, to the finger, fluctuating. The disease was plainly gaining ground, and rapidly; but still it was in an early stage, and in that which alone admitted of trying any remedy. Having determined these points, the only plan of treatment, he said, we could adopt was to remove the tumour by surgical operation; still the question remained, whether other parts were implicated in the same disease. As stated in the report, there was no glandular enlargement, or disturbed function in any of the viscera, to forbid an operation. It was then to be considered whether the tumour itself should be removed alone, or the arm. If the latter, it was obvious from the high point to which the disease reached in the axilla, that the limb must have been removed from the glenoid cavity of the scapula, or ex-articulated, as it is now the fashion to speak. With regard to the extirpation of the tumour, this was not thought practicable; for its extent was so great, and it was so involved with the muscles, that these and more important parts would have been divided before it could have been taken away, and great danger must have been anticipated from such an operation. It was also impossible to tell, from external examination, how far the disease actually extended, and, with certainty, to what parts it was attached. Another reason which particularly urged him to recommend amputation of the limb was, that he has never known any case, where the disease was removed from the part, that it did not there return. A lady, not more than 30, and in perfectly good health, became the subject of a tumour situated on the inner and upper part of the thigh (at the bottom of the superior triangle of which Poupart's ligament forms the base, with the apex below) the size of an orange when first discovered. It enlarged rapidly and soon increased to double that size; and, it appearing about to become malignant, Mr. Green recommended its removal without loss of time. Sir A. Cooper was called in, and agreed in Mr. G.'s view of the case, and in the propriety of the operation at that period. This was accordingly performed without delay. The tumour was attached to the sheath of the femoral vessels, behind which, it passed outwards, and also the origin of the adductor magnus muscle. However, the entire enlargement was completely removed, and both Sir A. Cooper and himself were satisfied that, if ever a local disease were wholly extirpated, it was in the present instance. Not the slightest doubt upon this point was in the mind of either; the tumour being in a firm capsule was easily turned out. The patient shortly recovered, and left town for the Summer to visit the seaside. On her return, after about four months, Mr. G. was again consulted, and sorry to perceive the disease had become re-established in the same part, and presented the same characters as before. In this patient, though in good health and young in years, the disease returned. Sir. A Cooper saw the case again, when the disease was rapidly advancing; amputation high up was thought of, but nothing save that at the hip-joint held out any prospect of relief. Ulceration took place, with frequent and profuse bleedings, and the patient sunk rapidly. This was so striking a case in the opinion of the lecturer, that he believed he need not recite more to shew the progress of some tumours, and their great danger when malignancy is suspected; and the circumstances of it weighed much in his mind when the case of Gooding presented itself.

Because we say the tumour was similar to pancreatic sarcoma, observed Mr. Green, you may tell me it was not fungoid disease, but this will take place in any kind of tumour, and from the rapid growth of late in the part, and other concomitant symptoms, we cannot say how soon it might have become so. Indeed, we can state that Mr. Travers expressed his firm conviction, from the section of the tumour, that it was of malignant nature. The whole mass was not cut into at the time of the clinical lecture, and Mr. Green, therefore, had not then seen the appearances mentioned towards the conclusion of our account of the dissection. In the first case related in this paper, that of Logan, we see that malignant action had been set up in a common absorbent glandular, or sarcomatous tumour; and even in an adipose tumour, Mr. Green has known fungoid action to take place. In the case of Gooding, as we believe fungoid action was commencing, it is of course doubtful whether this action will return, but Mr. Green has great hopes that the loss of the limb will save the patient, and that he will be able to enjoy many years of life free from disease. The tumour was not in the state of fungus, and his appearance is that of a man in the best health.

It has been observed that *torsion* of the arteries was effectual in restraining the hæmorrhage from two vessels in this case, and we are convinced the operation will come into general use. Mr. Green has trusted to it alone in a case of amputation of the breast lately performed, and also in the removal of a lip, and in both no bleeding followed. He stated in his lecture that Dr. Môlles, a Danish surgeon, who has lately visited this hospital, saw Amussat treat the *axillary artery* in the same way, in an amputation at the shoulder-joint, and the remedy was successful.

Upon the *after-treatment* of his patient, the lecturer remarked that the man was evidently suffering the temporary exhaustion after the operation from the shock of the same, he being more depressed than from the mere loss of blood. In point of fact, as he was operating upon a subject he might say in robust health, it was designed by him that some blood should be suffered to flow. He would not then give stimulants, which many might be tempted to do, as these were likely to bring on bleeding, and to increase the excitement afterwards. Touching the use of laudanum immediately after operations, he said he was not in the habit of ordering it, and in general saw no necessity for it; it might be injurious, increase fever, and constipate the bowels, which are the symptoms we would wish to obviate.

Our readers will no doubt feel interested to know the result of these cases, which will be of the greatest importance. We shall endeavour not to lose sight of the patients, and any further facts that come to our knowledge worthy of publication shall be recorded.

CASE 3.—*Tumours of the Breast.*

Clarissa Chamberlayne, æt. 26, admitted into Queen's Ward under the care of Mr. Green, Jan. 15, having three small tumours, very similar to scirrhus, in her left breast above the nipple. Two of these tumours are larger than the third, which is situated nearest to the axilla, the central one being the size of a walnut, and having been observed by her only four months. This has grown rapidly, having surpassed the next in size, which was noticed two months previously to it. The third is quite small, and the patient was unaware that it existed. At first they were unattended by pain, but this has been present during the last fortnight, of a darting and pricking kind, recurring at intervals. Tenderness is exhibited under firm pressure, but not otherwise. They are moveable with facility, and apparently connected to each other by a fibrous band running between them. The patient is a married woman, of delicate appearance, and what has been termed a leucophlegmatic habit. She has had two children, the younger of which is eighteen months old, and was weaned only four months ago, at the time the second and largest tumour was developed. Catamenia have been regular, and in proper quantity. She is free from dyspeptic symptoms, and her general health and spirits are unaffected, although she has never enjoyed strong health. Bowels are costive—the tongue clean and free from dryness—pulse rather quick. Ordered house medicine occasionally. Empl. saponis to the part.

Jan. 18th. Mr. Travers and Mr. Tyrrell have seen the patient, and do not seem to think the tumours are at present scirrhous. The former gentleman considered that they originated in simple adhesive inflammation of the lactiferous tubes, but observed that it was not safe to leave such tumours long to take their own course. He suggested the trial of iodine. Mr. Tyrrell said they possessed the precise feeling of scirrhus.

21st. The whole breast was to-day removed by Mr. Green, agreeably to the wishes of the patient, together with the tumours, by the usual operation. Some of the lactiferous tubes near to the axilla yielded a small quantity of milk on their division. Some of the bleeding vessels, which were very numerous, were twisted, but they were not of large size even for this situation, and the greater number ceased to bleed spontaneously. The edges of the wound were then approximated by dressing in the ordinary way. During the time the vessels were being twisted, and an examination of the wound was made to find out any more that might prove troublesome afterwards, the patient was rather faint and the pulse feeble.

Upon section of the two larger tumours, the structure appeared very similar in both. It was quite hard, internally of a whitish colour, and fibrous appearance, but had not the radiated surface we see in confirmed scirrhus. Nearly the whole internal section of the smaller tumour was of a pale colour; that of the larger one was peculiarly white in the centre, and light red, or mottled, towards the circumference. Externally to the central hardness, a thick yellowish fluid, not confined in any proper cavity, and very minute in quantity, appeared when an incision was made into the enlargement.

22d. Has passed a tolerably quiet night, and the pain, which continued in the wound for some time after it was dressed, ceased in the afternoon. Her pulse is, however, feeble, and 148—skin rather hot, but moist—tongue whitish and furred. No motion has passed from the bowels, but urine has been voided easily. There is no uneasiness now in the wound.

23d. There has been considerable pain in the wound for the last few hours, and rather copious oozing has taken place, which has excited much unnecessary alarm in the patient's mind. She slept well, and her bowels have been opened by a powder of scammony and calomel given yesterday. The pulse is 124, and feeble—tongue has a brown and dry streak along the centre to the tip, and is white at the margins—her thirst is less, and the skin not hotter. We removed the lint and some few of the straps, which relieved her of the uneasiness in the wound, by the exit of a reddish fluid (similar to venous blood, but thinner), which still was oozing freely. The edges of the wound appeared remarkably healthy, and were brought in contact again by the same means.

24th. The oozing has continued rather plentifully, but the patient is free from pain in the wound, or in any other part. She has passed a comfortable night, and her bowels are open. The pulse is very frequent, but regular —her tongue has lost its brown and arid streak—menstruation has been taking place during the last two days, and this no doubt accounts for the oozing from the wound, which possesses a peculiar character, being certainly similar in appearance and consistence to the menstrual fluid. The discharge from the uterus is more scanty than usual.

25th. Has been in no pain, either from wound, or in head; the pulse is at 110, and the skin naturally cool—her bowels are open, and tongue less white—wound has united throughout, except at its innermost point, and its edges are without the least redness.

27th. Patient quite free from pain and feverishness—wound was dressed, and a slight quantity of purulent matter escaped from the inner extremity—no oozing now takes place.

28th. The catamenia have ceased to flow, and there is a healthy discharge (purulent) from the wound, in the situation before-mentioned. This has united elsewhere, but the adhesions are not very firm, and require the support of strapping.

31st. No feverishness remains, and the wound continues united, affording a healthy discharge of pus at the one part only—pulse 90, soft, and natural in its beat—bowels open.

Feb. 2nd. Wound looks very healthy, there being only the one point ununited, and the patient is without complaint. Some hardness is felt passing from the incision towards the axilla.

7th. The granulations at the inner end of the incision are florid, and cicatrizing quickly—our patient is on house diet, and has rapidly regained strength and looks of health.

15th. Cicatrization is perfect, and the patient has recovered her former power—the hardness near the axilla is no longer to be felt.

17th. Presented well.

### IX. PURULENT OPHTHALMIA.

This form of ophthalmia is not frequently seen in this country in the adult, unconnected with gonorrhœa. The following case, however, appears to be a well marked instance of the disease in question.

Thomas Lester, æt. 23, a brewer's servant, accustomed to live comparatively temperately for this class of persons, was admitted under Mr. Travers's care on Thursday, the 4th November, 1830, having ophthalmia of both eyes attended with a most profuse purulent discharge. He stated that on the Monday week preceding he had gone some distance into the country on a job with his waggon, and returned home late at night, when he rubbed his eye (the right) rather roughly with his fingers, in consequence of itching and uneasiness in that organ. It then began to swell immediately; he, however, thinking the swelling would subside in the night, went to bed without taking further notice of the inconvenience he felt; but when morning came, the eyelids were so much swollen that he was unable to see with that eye. He says that it was not at all painful even then, nor has pain been present from the commencement. A copious discharge of what, from the patient's description, we should consider to be pus, at once took place. Treating his case lightly, he applied nothing to the eye but warm water fomentations, and sought no medical advice. In the course of a week the disease attacked the other eye in the same severe degree, except that there was not so much swelling of the palpebræ. The man expressly declares that at no period of his life has he had gonorrhœa, of which disease no traces are to be observed about the genitals, and he can give no other account than the above of the mode in which his eyes became affected. For our own parts, we are forced to the supposition, from what we can judge from present circumstances and what we have learned, that the inflammation of the conjunctiva arose simply from exposure to the night air, and was modified by some peculiarity in the atmosphere, or in the constitution of the patient.

At present slight pain only is felt in the left temple and brow, not very deep; both palpebræ superiores are greatly swollen, their texture thickened and somewhat indurated, the external integument of a dusky red colour, and their inner surface is tumid and of a brick red. The lower eyelids are in a similar state, though in a less degree. From this distention, the lids are of course approximated, all voluntary power over them being lost, and the upper one prolonged upon the cheek below the inferior tarsus; and as the patient is lying on his back in bed, a stream of white pus flows from the outer angles down the cheeks. The destruction in the right eye is much greater than in the left; vision is quite lost in this, the cornea being ulcerated, and some pus apparent in the anterior chamber. The conjunctiva is loaded with livid red vessels in a state of congestion. In the left eye, sight still remains, but far from perfect; the cornea is rather dull, and the conjunctival membrane is equally congested with the opposite. Slight chemosis has taken place on both sides, the cornea appearing depressed in consequence. The face is pallid—pulse not frequent, 84, and rather weak—skin cool—tongue whitish—appetite lost—bowels have not been regular.

Mr. Travers ordered the following to be injected into the right eye: argent. nitr. gr. iij. aquæ ℥j. M. To the left temple ten leeches to be applied, and to the left eye foment. papav. ℥j. aluminis, gr. iss, on linen constantly. Emplast. lyttæ nuchæ. ℞. Inf. cascarillæ, ℥x. Acid. sulph. dil. ♏v. Syrup. ʒj. bis die. Mist. sennæ comp. p. r. n.

Nov. 6th. Has no pain in the eyes, and there is not any perceptible alteration in their condition. Patient has slept well, and the bowels are opened by medicine.

7th. Some pain has been produced in the right eye since yesterday from the nitrate of silver solution, which deprived the patient of sleep last night, and has given rise to a degree of general feverishness. It is an unusual circumstance for pain to continue so long after the use of the nitrate. Pulse 80, more full—skin warm. He thinks the vision of the left eye is improved.

8th. Says he is much easier, and indeed in no pain; and the sight of the left eye has increased—drops of nitrate of silver omitted.

9th. Can to-day open the left eye. The cornea is clearer, and he can see more distinctly than at any former period since the attack. The redness and swelling of the upper lids have subsided, and are now confined to a red line at their margin.

11th. Somewhat improved since last date—discharge much less, and not so purulent; his appetite is very defective, and he does not recover strength. Quin. sulph. gr. j. Decoct. cinchon. ℥j. Acid. sulph. dil. ♏iij. Tinct. cardam. co. ʒss. Syrup. aurant. ʒj. M. 6tis horis sumend. Aluminis, gr. vj. Aquæ rosæ, ℥iv., to make a lotion, to be substituted for the fomentation.

14th. Not much alteration in the state of the eyes. Pulse is stronger, 90.

18th. Amendment progressive—discharge and redness of eyelids diminished. Remedies continued.

22d. Tumefaction and redness have quite abated from the palpebræ of the left eye. The cornea has one small speck of ulceration, situated a little above the axis of the pupil; but, excepting this, it is quite clear. The pupil is slightly oblong vertically, from adhesions formed anteriorly at its upper border; these, however, appear but trifling. It is contractile, and very little, if at all, diminished from the natural size.

30th. Cornea free from any opacity, and sight is nearly perfect; there is some irregularity of the pupil at its upper part, but it contracts and dilates nearly to the natural extent. There also exists a deposit of lymph on the anterior surface of the iris, above the pupil, connecting the iris to the cornea. The termination of the case is remarkably fortunate, and very different from what we are generally doomed to witness in this formidable disease. In the right eye there was not, at any period, the prospect of the restoration of vision.

Dec. 7th. Presented.

X. PHLEGMONOUS ERYSIPELAS.

Isabella Scrutching, æt. 12, residing at Somers Town, admitted under Mr. Green, December 7th, 1830. This girl is very tall and stout for her age, and has an unusually florid complexion, at present probably more than natural from flushing. Situated on the inner side of the left arm, from a little below the elbow-joint to the extent upwards of about four inches, the integuments are reddened for nearly the same space in the transverse direction of the limb, and are hot and tense; the part is also considerably swollen, and feels firm, indeed rather hard, and of course is painful under pressure. The inflammation commenced a fortnight ago, and the patient is unable to assign any cause for its appearance. A sharp and lancinating pain accompanies it, and the redness is diffused and of a dark hue, being uncircumscribed, and indicating that the subjacent cellular tissue is involved in the inflammation. Some of the axillary glands are enlarged and tender, and covered with a red surface. The constitution has not sympathised with this local disease as much as might be expected. The pulse is 100, not hard or full—skin quite cool—tongue rather dry and white—there is some head-ach, with nausea, but she has not vomited—bowels have been opened by medicine. Mr. Green made an incision, about two inches long, through the inflamed skin and cellular texture. It appeared to produce very considerable pain: very little blood was lost from the incision, and the bleeding was encouraged by fomentation; after this, the part was to be enveloped in a linseed poultice. The following medicines were prescribed; cal. gr. j. o. n. Magnes. sulph. ʒj. Vin. ant. tart. ♏xx. Mist. menth. ℥jss. 6tâ q. horâ.

Dec. 8th. Redness and tumefaction very perceptibly abated, and the integuments have lost their tense appearance. The patient has had a quiet night, with many hours' sleep—pulse is 100, soft, and rather small—tongue white—bowels have acted two or three times. Small portions of dead cellular membrane are loosely adhering to the edges of the incision, apparently detached from its lowest part; they are of a brownish colour, as well as the wound itself, and from the latter there is a slight discharge of pus.

9th. Inflammation subsiding—the redness occupies a less surface and is paler; there is rather a prominent and tender part on one side of the incision, as if from contained matter, but there is no softness, or fluctuating sensation. A bright rash has appeared since yesterday upon the arms, which commenced on the left around the wound, and is here now more conspicuous than in any other situation; it is continuous from the wrists to the shoulders, and is something like that of measles; the skin is of a dark red colour, and thickly interspersed with minute elevated spots, and, consequently, feels rough to the fingers. Some slight traces of it, as if quite in its commencement, are visible on the trunk, about the chest and scapulæ behind. It also exists on the thighs, but not nearly to the same extent as on the upper extremities. Her pulse is quicker than yesterday, but not sharp or hard. The efflorescence itself is hot, but the general suface is not elevated in temperature—tongue still white and dry—bowels very freely open; she experiences considerable itching and pricking pain from the eruption on the skin. The wound discharges but scantily, and looks as yesterday.

10th. Erysipelatous inflammation is gradually declining, and there is a more abundant discharge of pus, with some few shreds of cellular tissue in a dead state, from the wound. The rash on the skin has faded very considerably; the redness has nearly disappeared, together with the pimples, the remains of which still afford the same degree of roughness to the surface—prurigo is much diminished—the pulse soft, 100 —tongue less white.

11th. Redness of the skin no longer visible, except at the back part of the arm, opposite to the wound. The whole surface of the extremities is elsewhere of a natural colour, but still rough. There is no inflammation remaining around the wound, which has a favourable appearance, but the prominence near to it noticed on the 9th has increased, and a phlegmonous abscess would appear to be forming. She rests well — pulse is soft, 90 — tongue moist and clean — bowels continue to act freely.

13th. Skin is free from discolouration and roughness. The inflammation is quite subdued in the arm, and our little patient has no complaint to make. Some induration exists in the site of the former inflammation.

15th. No hardness now remains round the incision, which is healing by granulation, nor is there any swelling; and the appearances of abscess forming no longer remain. The child sits up the whole day, and appears in her general health as if nothing ailed her.

18th. Wound is strapped, and the patient on house diet.

28th. The wound is almost healed—ceratum calaminæ applied.

Jan. 13th. 1831. Presented well.

### XI. STONE IN THE FEMALE BLADDER —OPERATION.

Elizabeth Cook, æt. nine years, was admitted under Mr. Green, on January 25th, having symptoms of stone in the bladder.

Her mother states that, ever since the child was three years old she has observed some sandy deposit in her urine, mixed with what was considered to be mucus; but within the last three months only have decided symptoms of stone manifested themselves. During this time she has lost flesh rather rapidly, has suffered most acute pain, and been unable to leave her bed, her sufferings being greatly aggravated by exercise. Till the little patient attained her seventh year, she had been remarkably plump and healthy in appearance, though always but little disposed to exert herself with the same alacrity as other children of her own age, but the last two years her growth has been checked. About seven months ago four small calculi, described as being of very hard texture and resembling common pebbles, escaped from the bladder with her urine, but none have passed since. The poor child frequently screams out from pain, especially after the passage of urine, which she is generally not able to retain for any length of time, and scarcely ever in a quantity exceeding two ounces. Some of this fluid being collected in a glass measure appears very turbid, and deposits a thick sediment of mucus. Pain has existed in the loins, but not latterly; the most intense suffering is at present referred to the thighs. Her rest has been very much broken at night from obvious causes, and the countenance is anxious and worn. The appetite has not failed much; the bowels act regularly, and the motions are said to be healthy—pulse is nearly natural, and the tongue clean and moist. Of late she has taken considerable doses of opium in the solid form, as often as four or five times a day, which remedy she has borne in a manner unusual for children so young; and the parents have also been in the habit of giving her gin.

Jan. 27th. She has been in extreme pain, evinced by violent screams, but got some few hours uninterrupted sleep last night. In the evening she had pain at the lowest part of the abdomen, but no tenderness. The former exists to-day, and leeches, with fomentations, were applied. The little sufferer endeavours to obtain ease by sitting up in bed with her thighs approximated to the abdomen. On testing the urine, it is found neutral. Three grains of ext. hyoscyami were given at night.

28th. Passed a good night, but has been in the greatest tortures the whole morning. Her cries are attended by strong action of the respiratory muscles, and the mother informs us that a hernial protrusion has taken place on the left side, from the abdominal ring, and most probably of the direct kind, but none has been observed since she has been here. About three o'clock in the afternoon, as she was in so great agony, Mr. Tyrrell was requested to see her. He refrained from putting the patient to the pain of introducing a sound, as Mr. Green would see her in the evening, and would probably then pass one into the bladder. Mr. T. directed her to be placed in a warm bath; and five minims of liquor potassæ with five of liq. opii. sedat. to be exhibited every two hours till relief of pain was obtained.

9 o'clock, p. m. Has taken three doses of the medicine, and has been easy since eight; medicine to be administered only when required by pain in the night.

29th. In consequence of the pain recurring last night, the dose had been repeated four times by twelve o'clock to-day. Mr. Green now sounded her and discovered a calculus of large size, and apparently of the mulberry species, in the bladder. The hernia was noticed during the struggles of the child, issuing from the external ring. Medicine to be omitted.

30th. Has been somewhat easier—her stools are white, but pulpy—Mr. R. Whitfield ordered a dose of ol. ricini.

31st. Was placed in the bath early this morning on account of the severity of pain, and it was speedily effectual in relieving her. Has had a stool more healthy in colour.

Feb. 1st. Mr. Green to-day removed the calculus from the bladder by an operation, which consisted in cutting through the urethra and neck of the bladder with the gorget, passed along the staff (the groove of which was directed downwards,) with its sharp edge turned outwards and a little downwards. The urine escaped with great force when the bladder was cut into. The stone, which was larger than a walnut, and somewhat of the same figure, weighing ℥iv. ℈ijss., and of the oxalate of lime composition, was easily felt and laid hold of by the forceps. But these, being of small size, slipped from the surface of the calculus owing to their want of capacity to retain their hold; a larger pair being introduced, it was forthwith extracted. Not an ounce of blood flowed from the wound. The child was very irritable and struggled much during the operation, which gave rise to descent of the hernia and protrusion of the rectum. After the operation her countenance was sunken and anxious, and she was crying from pain for above an hour. She continued to suffer in a less degree throughout the afternoon, and in the evening at 10 o'clock we found she had passed urine freely through the wound, no hæmorrhage had taken place, but she had suffered much uneasiness in the pubic region. No tenderness here existed, and her breathing was tranquil. Pulse rather quick, 112; skin quite temperate.

2d. Her night has been disturbed, she having slept very little, but she has had neither pain nor tenderness of abdomen. A copious liquid stool of a natural colour has just come away, and the pulse is now little above 90, and natural in its beat; in the space of an hour, however, it returned to 112—skin cool. The urine has flowed without interruption.

*Vespere*. Bowels have acted twice since morning; she has had some little abdominal pain. The pulse is the same, but compressible.

3d. Owing to the recurrence of rather severe pain last night, about 3 o'clock, leeches were applied to the hypogastrium, and have produced most immediate relief. Her pulse is tranquil, and skin cool, and she slept for some hours after the local depletion of blood. Countenance placid, and has lost all expression of anxiety. Flow of urine unobstructed.

*Vespere*. Has been quite free from uneasiness, and has retained her water for an hour. Some superficial redness has existed on the nates for an hour or two, but it is not inflammatory in character—bowels open.

4th. The redness noticed last night has quite disappeared, and was produced simply by the irritation of the urine. She has slept well, and the pulse is 108, soft, and compressible—tongue is clean and moist. Her water was retained two hours together in the night. One stool. Allowed bread pudding.

5th Has passed a good night and is in every respect well.

6th. No unfavourable symptom to be mentioned.

7th. Patient well—urine is now constantly flowing; pulse 110; it has varied from time to time, but has never been under this standard—bowels have acted regularly every day—and the evacuations are healthy. She partook of some chicken yesterday.

9th. Wound has been healing most favourably; the little patient has taken meat, and continues to rest well. Her pulse is ranging from 110 to 130.

12th. In every respect well.

15th. Retains her urine for three or four hours at a time.

22d. The little patient has not been allowed to leave her bed yet, but may be considered as perfectly recovered from the effects of the operation, having no incontinence of urine. Her pulse is frequent, but in a child this is a matter of little importance.

After having read the above case to the class, Mr. Green observed in a clinical lecture that the reasons which induced him to prefer the operation of cutting into the bladder to that of dilatation of the meatus urinarius, now generally adopted in the female, were these. In children it is very difficult to keep the body perfectly quiet, whilst the dilatation is being effected and the calculus brought through the canal. This patient was obviously excessively irritable; and a longer period would be necessary for dilating the urethra, and more distress altogether produced than from the simple incision that was made. As it afterwards appeared too, the calculus was too large to be abstracted with safety by any other mode.

### XII. INTERESTING CASE OF LITHOTOMY.

John Wilkinson, æt. 19, by occupation a chair-maker, was admitted by Mr. Green, Feb. 3d, 1831, from High Wycomb, Buckinghamshire. About six years ago, we are informed, he had some symptoms of stone in the bladder, such as pain in the region of this viscus and in the penis, and the passage of blood with his urine; and from these he has not been free during this period, although they have been productive of little or no uneasiness up to the last six weeks. A greater degree of pain having latterly come on without any assignable cause, he was sounded by a surgeon in the country, and a calculus detected a fortnight since. The patient says that on Christmas night he was first attacked with severe pain, which has since recurred at short intervals and become more intense. His urine is clear, and he is devoid of pain in the loins; such did exist three or four years back. At night his rest is disturbed, he being often obliged to make water eight or ten times during the period he is in bed, but his general health is not affected, the pulse being 70, and the tongue clean. There is slight constipation of the bowels. He has been addicted to drinking rather in excess, and the face is thickly covered with black pimples, the complexion being dingy and unpleasant to the sight, and of an unusual leaden hue.

Feb. 8. Was sounded to-day by Mr. Green, and a calculus found, situated over the vesical extremity of the urethra, so that it was instantly struck by the instrument. It appeared to Mr. Green to be very rough, and probably of the mulberry kind. Patient's bowels are regulated by the house-medicine, and he makes no complaint other than what is referrible to the above obvious source. He is free from cough and every other symptom of pulmonary disorder, and his urine is quite transparent, and slightly acidulous. The face is not, however, clearer from the black spots.

11th. The operation of lithotomy was this day performed with the instruments used by Mr. Green, viz. a knife, to make the incisions down to the staff, and a gorget, wherewith to cut into the bladder. When the calculus was grasped with the forceps, it was found to be of larger size than was anticipated, and its surface very irregular. Some attempts were made by the operator to remove it with the instrument, but this being found impracticable, the internal wound was dilated with a bistoury, after which the calculus was withdrawn by means of a larger pair of forceps. Rather profuse hæmorrhage occurred from the transverse artery of the perineum, which was pouring forth blood during the whole operation, and this lasted upwards of 20 minutes. The difficulty in the extraction arose from the size and nearly spherical figure of the stone; and it was also increased by the unusually rough (or aculiate) surface of the latter, which we might say was studded with pointed excrescences of nearly a sixth of an inch in length.

When the operation was concluded, the patient was a little depressed and felt thirsty; but his pulse was good, and all bleeding had ceased with the removal of the concretion. He complained of pain in the situation of the bladder for some hours, was rather restless, and wished to change his position, from the back to that on his side. At nine o'clock in the evening we learned that slight pain had frequently recurred in the hypogastrium, and that half an hour before a large quantity of coagula, with some fluid arterial blood, had escaped from the wound. He was now easier, but felt considerable depression and thirst—pulse 90, quite soft—no tenderness of abdomen—urine had flowed freely through the wound, and a small quantity from the penis, which often happens soon after the operation from the presence of coagulated blood between the urethra and external wound. Dry fomentations were applied. Feb. 12th, 10, a. m. Has slept at intervals during the night, and feels less depression—his water has escaped in abundance. There has existed some permanent pain, with tenderness, in the left iliac region for the last two hours —pulse 110, soft—skin cool—tongue rather white. Twelve leeches were applied to the part alluded to by the direction of the dresser.

Feb. 12th, 4, p. m. The pain and tenderness were relieved by the leeches, but the pulse has been progressively increasing in force and frequency; it is now at 140, irregular and hard. The respiration has been but little affected. Tenderness seems more diffused, but is less severe—skin moist and temperate. Soon after the leeches were removed, he was chilled and felt an internal coldness, which has ceased, and at present there is a sensation of heat internally; some headache has been present, with a constant craving for drink. A linseed poultice has been applied over the leech-bites. Mr. Green had a vein opened in the arm, and about 18 ounces of blood being taken, the heart's action ceased. When this had returned, the pulse had lost its hardness and was less frequent. Mr. G. directed ʒij. of ung. hydr. to be rubbed into the thighs after the elapse of an hour, when the following pills were also to be commenced every four hours. ℞. Hydr. submur. gr. iv. Pulv. opii, gr. ¼, M. Should any pain continue in the hypogastrium, 20 leeches to be re-applied, and if after six hours the pulse should remain hard, the mercurial friction to be then repeated.

11, p.m. Some pain and tenderness have remained till now in the hypogastric region; the pulse is 140, rather jerking, but regular, and admitting of compression—skin cool, but not moist —headache has ceased. The rubbing in was accomplished without any inconvenience, and a second relay of leeches was put on about nine o'clock. A glyster of gruel and castor-oil has just been administered. It was not thought necessary to repeat the friction, the dresser judging from the altered state of the pulse.

13th, 9, a.m. He has enjoyed some sleep in the night, and had but slight pain, which still continues, along with tenderness, but both are much less intense than last evening—pulse 126, irregular in force and beat, softer, and not jerking—respiration natural. A copious stool followed the exhibition of the injection, and almost instantly afterwards there was very marked relief. A light bran poultice has been kept on the belly—tongue whitish, moist—skin also moist. Yesterday evening a saline mixture, containing the liq. ammon. acetat. and the tartrite of antimony was given every four hours; but having produced sickness, it is now omitted. The countenance is of much better expression, and thirst has abated. Has taken the calomel and opium with regularity, and these are continued.

3 o'clock, p. m. Pain and tenderness under pressure have been gradually decreasing, and the man has slept occasionally. Pulse 118, regular, full, and soft—redness of the gums is produced, with the mercurial fetor and taste—

urine has flowed freely, and he has had another evacuation from the bowels. Mr. Green visited him at eleven o'clock, and ordered two drachms more of unguent. hyd. to be rubbed in immediately, and four grains of calomel with one of opium to be taken every six hours. Arrow-root and rice are allowed, and if thought necessary, beef-tea.

10, p m. The countenance has very much improved since morning. The abdominal pain and tenderness continue, so that twenty more leeches have just been applied Pulse 118, full, and soft—sickness and headache have quite left—breathing performed with natural facility—gums have become more sore, and the bowels have been open twice since three o'clock. The dresser desired half the quantity of calomel to be now administered every sixth hour.

14th, 9, a.m. Much less pain exists, and he bears tolerably firm pressure—has been quite easy throughout the night. Pulse 116, in the same condition as last mentioned—urine has passed in abundance. There is some swelling of the jaws from the mercury, which is ordered to be intermitted until Mr. Green's visit. No motion from the bowels.

10, p.m. At two o'clock Mr. Green saw him, and found the pulse at 120, full, but compressible. A motion had just passed, preceded by griping, though solid. He ordered the friction to be again employed, preferring this to giving the submuriate by the mouth in the present irritable state of bowels, and an injection of two ounces of starch and 40 minims of laudanum to be exhibited immediately. Shortly after it was thrown up a state of slight collapse came on, attended with nausea and faintness; these have, however, ceased, after taking a few ounces of the effervescent mixture. The pain and tenderness of abdomen have been gradually diminishing, and the pulse is reduced to 100 and is less full. Two more stools have been passed, with griping pain, and in one of them a large lumbricus was voided. A grain of opium was given by the dresser, in soap, and beef-tea directed to be exhibited in the night should he appear to get weaker—and, if requisite, ammon. carb. gr. vj. every fourth hour.

15th. 10, a.m. A state of depression again occurred at three o'clock this morning, with frequent purging of scanty stools, and he has enjoyed no rest. The ammonia has accordingly been taken, and at present he is revived and disposed to sleep. Complains of pain near the umbilicus from griping, but has not inflammatory pain or tenderness, and an evacuation came away about half an hour ago. Pulse 98, regular, not so full or strong—mouth very sore—tongue moist has a whitish fur over the whole dorsum—countenance is rather sunken. The urine has been voided in sufficient quantity, and principally through the urethra; wound looks healthy, and there is no surrounding redness, nor excoriation. A glyster of starch and laudanum was ordered to be again injected, and another dose of the ammonia immediately, the last having been taken at 7 o'clock. His linen and sheets have been changed, from which he has derived great comfort.

8, p.m. At Mr. Green's visit, at two o'clock, he found pretty firm pressure borne in every part of the abdomen without the least pain, and no unfavourable change since morning was observable. Two motions had, however come from the bowels, and, therefore, the enema was repeated; the ammonia, &c. to be discontinued. He has slept for some hours during the day; and at present the pulse is 110, and rather more powerful, and there has been no recurrence of pain or stool since the injection.

16th. 10, a.m. During the night the bowels have been purged four or five times, and acute tormina have attended each evacuation, but there is no tenderness of belly, nor permanent pain. Owing to this state of bowels no rest has been obtained—the pulse is 110, not full, nor strong—tongue more thickly coated with a white fur, and gums more affected by the mercury. Urine has been voided in greater quantity through the natural passage, and the wound is looking well. Mist. cretæ co. ℥iss. 4tis horis. Mouth is washed with a gargle of chloride of soda.

9, p.m. Bowels continue equally relaxed—pulse 118, stronger. He has taken rice-water and milk, which latter, he says, was accustomed to purge him when in health. Mr. Green gave orders that the following mixture should be given every four hours. ℞. Magn. sulph. ℈ij. Tinct. opii, ♏v. Mist. menth. ℥jss.; and, if this do not effect some tranquillity of the bowels after the first few doses, ℥ij. of the compound infusion of catechu to be substituted; omit milk.

17th. Several motions have passed during the night, but they have been less copious, and the patient's bowels have been quieter, and not in their former uneasy state. A dose of the catechu was supplied at five o'clock this morning, and two or three evacuations, not accompanied by pain, have since taken place. No pain nor tenderness continues; the pulse is 100, and soft—tongue coated thickly; but, as the mouth is very sore from mercury, it probably originates from that cause. Urine has passed freely, both by wound and urethra. To have some bread pudding, and the catechu to be repeated, if required by the state of the bowels.

18th. A dose of the catechu was given last night, and the bowels have been open several times, but not since five o'clock—pulse 96, nearly natural—mouth less sore, from the use of a gargle of chloride of lime—rested well last night—urine uninterrupted—countenance almost as before the operation. To have bread pudding, &c.

19th. Has slept well, and his pulse is at 88—gums very sore, and the under surface of the tongue is ulcerated. One scanty motion was discharged at five, and none subsequently.

20th. Pulse 88, natural in strength—ulceration of tongue and mouth diminished, under the frequent use of the chloride of lime gargle—no stool has been voided since yesterday. Patient suffers nought but from the state of his mouth, but was able to eat some chicken.

22d. Feels quite strong; and, were it not for his mouth, he says he would not have cause for complaining. A little meat allowed; bowels not purged.

24th. A very little water passes through the wound, and he is able to empty the bladder voluntarily. The bowels have not acted since the 22d, and a dose of mist. sennæ comp. has been this morning given.

26th. Bowels were opened several times by the medicine; the patient's mouth is still so sore as to prevent his masticating meat; but, with this exception, he complains of nothing; pulse 84, of nearly natural beat. He takes a little porter, and has an excellent appetite.

The power of mercury in subduing inflammation, of membranous surfaces in particular, is now well known; but, under circumstances similar to the present, Mr. Green had seen it employed in two cases only, viz. of strangulated hernia, where inflammation of the peritoneum had come on, being complicated with that of other textures in the vicinity of the wound. In both these instances, the effects of the remedy were as decisive as in the purest idiopathic inflammation. It was evident, from the symptoms which became developed on Feb. 12th, in the above case of lithotomy, that very serious inflammation of the peritoneal membrane had set in, originating probably in the cellular tissue between the latter and the organs contained in the pelvis, which was of course wounded in the operation, and, when inflamed, is very prone to *sloughing*. Mr. Green considered that the patient, whose circulation during health had been languid and deficient in energy, as evinced by the state of countenance, would not bear much further depletion by venesection; yet some active treatment was required. Judging from the great utility of the plan in ordinary instances of phlegmasia, and the favorable results in the two cases of hernia, he, therefore, determined upon the trial of mercury, to be given to affect the mouth speedily.

It might be said that the patient would have done well without the mercurial treatment; but Mr. Green does not think this at all a probable view of the case, for, in his opinion, the inflammation would have proceeded, (and who can tell to what extent?) and proved destructive to life. The issue of the case was to him most satisfactory, and such, that he would be strongly disposed to adopt the same means in future under like circumstances.—BETA.

